# Descriptor-Guided Design of Mo-Doped FeCoNiCu High-Entropy Alloy Electrocatalysts Surpassing Pt for Alkaline Hydrogen Evolution

**DOI:** 10.1021/acsami.5c13488

**Published:** 2025-09-11

**Authors:** Shiqi Wang, Haixian Yan, Wenyi Huo, Mahmoud Abdellatief, Feng Fang, Pedro H. C. Camargo

**Affiliations:** † Jiangsu Key Laboratory of Advanced Metallic Materials, 12579 Southeast University, Nanjing 211189, P. R. China; ‡ Department of Chemistry, 3835 University of Helsinki, A.I. Virtasen aukio 1, PO Box 55, Helsinki FIN-0014, Finland; § College of Mechanical and Electrical Engineering, 74584 Nanjing Forestry University, Nanjing 210037, P. R. China; ∥ NOMATEN Centre of Excellence, National Centre for Nuclear Research, Otwock 05-400, Poland; ⊥ Synchrotron-light for Experimental Science and Applications in the Middle East (SESAME), Allan 19252, Jordan

**Keywords:** high-entropy alloys (HEAs), hydrogen evolution reaction (HER), multidescriptor computational screening, DFT calculations, bifunctional water splitting

## Abstract

High-entropy alloys (HEAs) offer an immense compositional playground for electrocatalyst discovery. Yet, the rational navigation of this space remains elusive. Here, we introduce a multidescriptor screening strategy combining density functional theory (DFT) calculations and data analytics based on critical parameters including d-band position, water dissociation energetics, hydrogen adsorption free energies, lattice stability, and corrosion resistance. This methodology systematically evaluates FeCoNiCu-based HEAs doped with transition metals (Ti, V, Cr, Zr, Nb, Mo, and W), identifying Mo as the optimal dopant due to its ideal balance between a low water dissociation barrier (0.41 eV) and near-thermoneutral hydrogen adsorption energies at Fe–Co–Ni hollow sites. Guided by computational predictions, phase-pure Mo-rich FeCoNiCu HEA films synthesized via magnetron sputtering deliver outstanding alkaline hydrogen evolution reaction (HER) activity, with an overpotential of just 60.1 mV at 10 mA cm^–2^, exceptional durability at −200 mA cm^–2^ over 100 h, and performance superior to commercial Pt/C catalysts. Soft X-ray absorption spectroscopy reveals dynamic Mo-mediated electron transfer among Fe, Co, and Ni, facilitating a dual-site Volmer–Heyrovsky mechanism. This study not only establishes an earth-abundant HEA that eclipses Pt for alkaline HER but also showcases a scalable “compute-screen-make-test” paradigm that can accelerate electrocatalyst discovery across the vast HEA design space.

## Graphical abstract

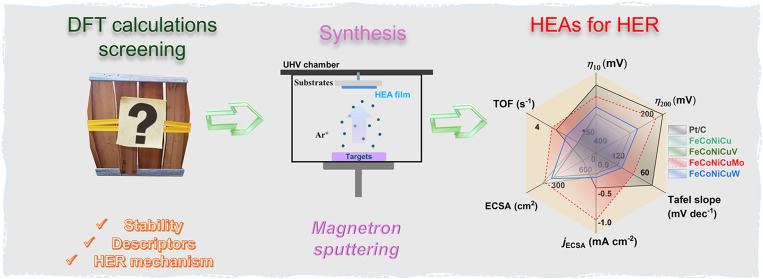

## 1 Introduction

Green hydrogen produced by water electrolysis is rapidly becoming a cornerstone of the low-carbon energy economy.^1,2^ Among commercial electrolyzer platforms, the alkaline water electrolysis (AWE) is uniquely attractive because it (i) scales readily to the multimegawatt level, (ii) tolerates inexpensive, earth-abundant electrodes, and (iii) avoids the acidic,^3–5^ precious-metal-intensive environment of proton-exchange-membrane (PEM) systems. The main roadblock is kinetic: in alkaline media, the hydrogen evolution reaction (HER) is slowed by an extra water dissociation (Volmer) step, raising the overpotential and eroding system efficiency.^6,7^ Catalysts that accelerate this step without sacrificing durability are therefore essential.

High-entropy alloys (HEAs), metallic solid solutions comprising five or more principal elements, have emerged as a disruptive answer to that need.^8–10^ Their severe lattice distortion, sluggish diffusion, and rich palette of surface motifs offer an unparalleled platform for tuning adsorption energies and corrosion resistance.^10^ In particular, FeCoNiCu-based HEAs combine low cost with electronic structures well suited to hydrogen electrocatalysis.^11,12^ Yet the same compositional freedom that makes HEAs exciting also makes them daunting: conventional trial-and-error synthesis cannot hope to navigate millions of possible formulations, and existing data-driven studies rarely target alkaline HER requirements.^13,14^

Here, we close that gap by marrying multidescriptor density functional-theory (DFT) screening with targeted experiments. We systematically evaluated the quinary series FeCoNiCuM (M = Ti, V, Cr, Zr, Nb, Mo, W, which remain insufficiently investigated) using key descriptors, including d-band center (*d*_c_), water- and hydroxide-adsorption energies, hydrogen binding free energy, and dissolution potential, to identify candidates that balance fast Volmer kinetics with long-term stability.^15,16^ The analysis singled out Mo- and W-doped alloys as optimal. Guided by these predictions, we fabricated single-phase FeCoNiCu, FeCoNiCuV, FeCoNiCuMo, and FeCoNiCuW thin films via magnetron sputtering. FeCoNiCuMo outperformed commercial Pt/C, delivering 60 mV at 10 mA cm^–2^ and sustaining −200 mA cm^–2^ for 100 h with negligible degradation. This integrated “compute–screen–make–test” workflow provides a practical map through the vast HEA compositional space, delivers the first earth-abundant HEA that eclipses Pt for alkaline HER, and establishes a general blueprint for accelerating discovery of multielement electrocatalysts for sustainable energy technologies.

## 2 Results and Discussion

### 2.1 Data-Driven Computational Screening Strategy

The rational design of high-performance FeCoNiCuM HEA catalysts for the alkaline HER was guided by a data-driven computational screening strategy (Figure 1A). This integrated workflow combined first-principles DFT calculations, with spin polarization and dispersion corrections, with composition-controlled synthesis via magnetron sputtering (precision ± 1 atom %) and subsequent experimental validation. The primary objective was to identify suitable transition metal dopants (M = Ti, V, Cr, Zr, Nb, Mo, W) that, when alloyed into an FeCoNiCu host matrix, could enhance both the thermodynamic stability and the electronic structure of the resulting quinary HEAs, thereby promoting efficient alkaline HER catalysis. Key descriptors such as surface stability, H_2_O adsorption energy, OH^–^ desorption energy, and hydrogen adsorption free energy (Δ*G*_H*_) were systematically computed to guide the selection of optimal compositions (see Experimental Section and Supporting Information for computational details).

**Figure 1:**
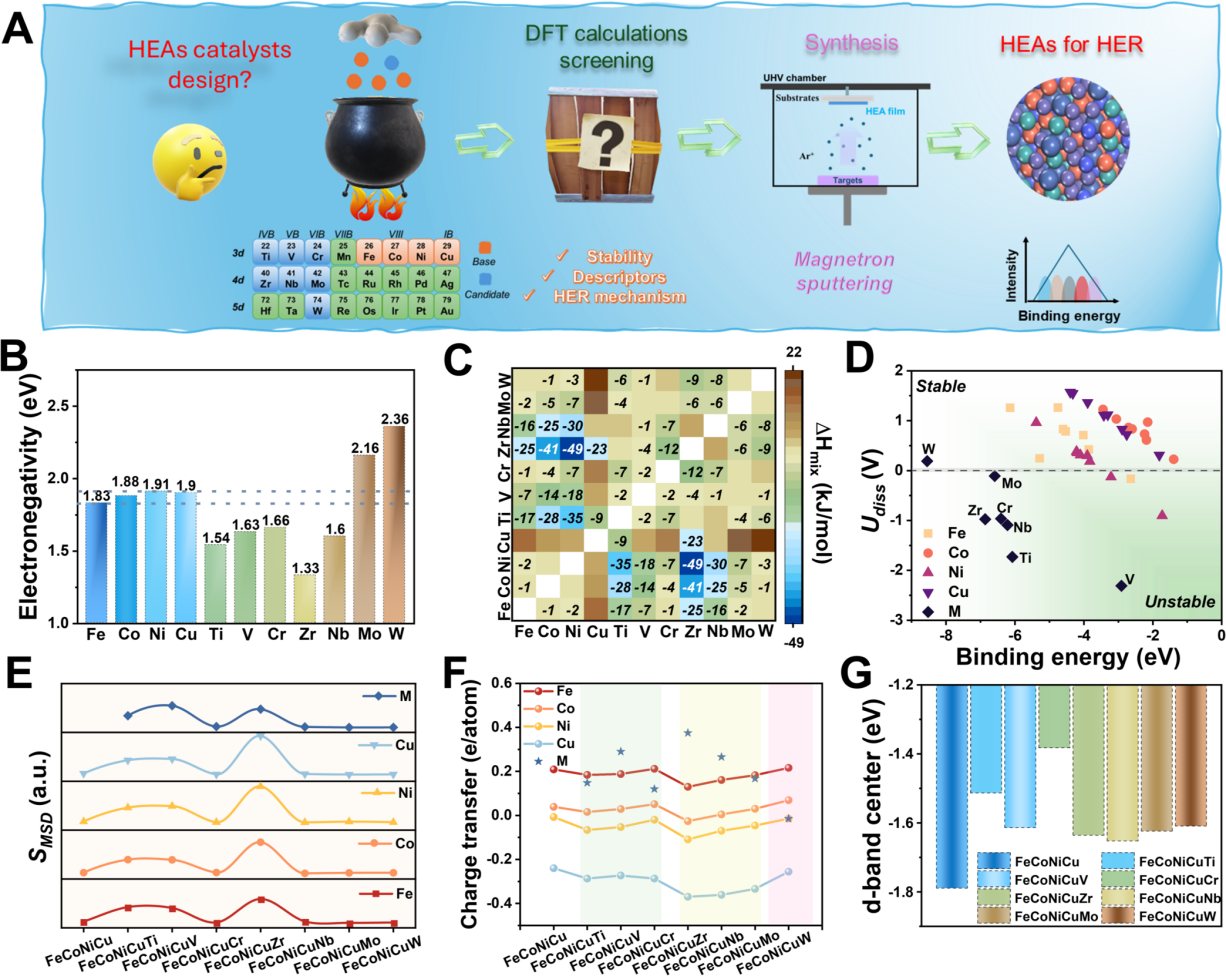
Computational design and screening of FeCoNiCuM HEA catalysts for alkaline HER. (A) Integrated workflow combining DFT-based screening, composition-controlled synthesis, and electrochemical evaluation. (B) Pauling electronegativities of constituent elements. (C) Binary mixing enthalpy (Δ*H*_mix_) matrix, where near-zero or negative values favor the formation of homogeneous solid solutions. (D) Stability map correlating site-specific binding energies (BE) and dissolution potentials (*U*_diss_). (E) Mean square displacement (*S*_MSD_) from ab initio molecular dynamics simulations. (F) Charge transfer distributions across HEA models. (G) Calculated *d*_c_ values for FeCoNiCuM alloys.

To rationally narrow the compositional space, several randomized FeCoNiCuM surface configurations were first screened using fundamental thermodynamic and structural stability descriptors:^17–19^ atomic radius mismatch (Δ*r*), Pauling electronegativity difference (Δχ), and binary mixing enthalpy (Δ*H*_mix_) (Figures S1–S8). The atomic radius mismatch is a key factor influencing lattice strain and phase stability in HEAs. Elements such as Ti, Zr, and Nb, which exhibit substantial size mismatches (Δ*r* > 15%, Table S1), were deprioritized due to their propensity to induce local distortions and structural instability.^20^ Electronegativity, another critical descriptor, governs charge transfer and bonding characteristics.^17^ The host matrix elements (Fe, Co, Ni, and Cu) feature closely matched electronegativities (1.83–1.91; Figure 1B), facilitating electronic homogeneity. The introduction of elements with higher electronegativities, such as Mo (2.16) and W (2.36), enables targeted modulation of the electronic structure and surface charge distribution, which can enhance corrosion resistance and improve the adsorption energetics of HER intermediates. However, excessively large Δχ values may promote phase segregation rather than the formation of a uniform solid solution.^21^ Mo and W provide a favorable balance, introducing moderate electronegativity contrast without destabilizing the alloy matrix. Complementing these electronic considerations, binary mixing enthalpy (Δ*H*_mix_) offers a thermodynamic lens into phase formation tendencies (Figure 1C and Table S2). Ideally, mildly negative or near-zero Δ*H*_mix_ values favor solid solution formation and suppress intermetallic compound formation.^21^ Pairs such as Fe–Mo (−2 kJ mol^–1^), Co–Mo (−5 kJ mol^–1^), and Ni–Mo (−7 kJ mol^–1^), as well as Fe–W (0 kJ mol^–1^), Co–W (−1 kJ mol^–1^), and Ni–W (−3 kJ mol^–1^), all satisfy these criteria, underscoring the thermodynamic viability of incorporating Mo and W into the FeCoNiCu matrix.

To further evaluate the structural and electrochemical stability of the FeCoNiCuM HEAs, we examined key energetic and dynamic descriptors: binding energy (BE), dissolution potential (*U*_diss_), and mean square displacement (*S*_MSD_) derived from molecular dynamics (MD) simulations. BE serves as a proxy for the cohesive strength of atoms within the alloy matrix; more negative BE values correspond to stronger local bonding.^22,23^ Incorporation of Cr, Mo, and W generally increased the magnitude of BE for the Fe, Co, Ni, and Cu sites, indicating enhanced atomic interactions and greater local structural integrity (Figure 1D and Table S3). Electrochemical robustness was further assessed via *U*_diss_, where positive values signal resistance to oxidative dissolution in alkaline media. The calculated *U*_diss_ values (Figure 1D and Table S4) confirm that Fe, Co, Ni, and Cu in most FeCoNiCuM compositions remain stable under HER-relevant conditions, with FeCoNiCuV showing slightly diminished stability at select sites. In contrast, the fifth elements, particularly Ti, V, Zr, Nb, and Mo, tended to exhibit negative *U*_diss_ values, reflecting a higher susceptibility to leaching.^22^ Notably, W was an exception, exhibiting a slightly positive *U*_diss_ (0.189 V), thereby offering a unique balance of the bonding strength and electrochemical stability. MD simulations were employed to probe atomic mobility and thermodynamic stability through calculation of the *S*_MSD_. Lower *S*_MSD_ values correspond to reduced atomic diffusion, and thus greater structural stability over time.^9^ As illustrated in Figure 1E and Table S5, the incorporation of Ti, V, and Zr into the FeCoNiCu matrix generally increased the *S*_MSD_ values of the constituent atoms, signaling less stable and more dynamically disordered configurations. In contrast, Mo and W either preserved or reduced the *S*_MSD_ of the host elements, indicating their favorable role in suppressing atomic diffusion and enhancing lattice cohesion.^24^ These trends underscore the capacity of Mo and W to improve the overall structural integrity of FeCoNiCu-based HEAs. Importantly, these elements are also recognized as oxophilic transition metals, a feature that facilitates the Volmer water dissociation step while simultaneously enabling the fine-tuning of hydrogen adsorption energetics, both of which are critical for efficient alkaline HER. Taken together with binding energy and dissolution potential analyses, these findings identify Mo and W as optimal dopants for simultaneously improving both thermodynamic and electrochemical stability.

Beyond structural stability, the electronic structure of HEA catalysts plays a critical role in governing the catalytic performance. To this end, we evaluated several key electronic descriptors, including charge transfer, charge density difference (CDD), electron localization function (ELF), work function (WF), projected density of states (PDOS), and the *d*_c_. Charge transfer analysis (Figure 1F and Table S6) reveals pronounced electronic redistribution upon the incorporation of the fifth transition metal, confirming the formation of hybridized metal–metal bonds and strong interatomic interactions.^23^ Notably, Cu consistently exhibits a net negative charge across all models (e.g., −0.24 e/atom in FeCoNiCu), a feature that could facilitate *OH desorption, a known kinetic bottleneck in alkaline HER.^25^ Doping with Zr, Nb, and Mo further modulates the electronic environment at Cu sites; for instance, Mo incorporation increases the electron density on Cu (−0.334 e/atom in FeCoNiCuMo), indicating enhanced electronic polarization that may benefit intermediate binding and desorption dynamics.^25^ This effect is visually corroborated by 2D-CDD maps (Figure S9), which depict regions of electron accumulation and depletion induced by the dopant atoms. ELF analysis (Figure S10) further suggests that Mo, Zr, and Nb enhance electron localization at specific lattice sites, while promoting electron delocalization across neighboring Fe, Co, and Ni atoms, an interplay that may stabilize key HER intermediates.^26^ Consistent with these trends, planar-averaged differential charge density (DCD) profiles (Figure S11) reveal dopant-induced modulation of the charge distribution across the surface, potentially improving both electrical conductivity and surface reactivity.^9^

The work function (*W*_F_) of the catalyst surface is a critical descriptor of its electron transfer capabilities.^27^ Compared to pristine FeCoNiCu (*W*_F_ = 4.08 eV), doping with Ti or Mo significantly lowers the *W*_F_, FeCoNiCuTi (3.03 eV) and FeCoNiCuMo (3.41 eV) (Figure S12), thereby facilitating electron donation to adsorbed species and enhancing HER activity. A more nuanced view of electronic structure is provided by the projected density of states (PDOS), where the d-band center (*d*_c_) serves as a key indicator of adsorption strength.^28^ In general, a d-band center closer to the Fermi level (*E*_F_) correlates with stronger adsorbate–metal interactions. As shown in Figures S13 and S14 and summarized in Figure 1G and Table S7, most FeCoNiCuM compositions exhibit an upward shift in *d*_c_ relative to FeCoNiCu, suggesting enhanced electronic activity.^28^ Among these, FeCoNiCuCr displays the most positive shift. However, overly elevated *d*_c_ values can lead to overbinding of intermediates and surface poisoning, underscoring the importance of achieving a balanced shift.^23^

To dissect element-specific contributions, site-resolved PDOS and *d*_c_ analyses were performed (Figures S13, S15–S19). The Fe 3d orbitals (Figure S15) exhibit *e*_g_–*t*_2g_ splitting due to local distortions and ligand field effects, promoting d–d orbital hybridization.^9^ In most quinary HEAs, Fe *d*_c_ shifts closer to *E*_F_, favoring stronger intermediate binding. Co and Ni 3d states (Figures S16 and S17) lie moderately below the E_F_ with broad distributions, which are crucial for optimizing adsorption energetics. Interestingly, the addition of a fifth element often shifts Co *d*_c_ upward and Ni *d*_c_ downward, reflecting their distinct roles in modulating reactivity. Cu 3d states (Figure S18) remain well below *E*_F_ across all models, functioning as electron reservoirs.^8^ In particular, the Cu *d*_c_ in FeCoNiCuMo (−3.16 eV) is significantly downshifted, a feature that may reduce *OH overbinding and improve catalytic durability.^29^

Dopant-site PDOS profiles (Figure S19) further reveal that Ti, V, and Cr possess narrow d-bands, while Nb, Mo, and W show broader d-bands extending across *E*_F_, indicative of stronger d–d orbital overlap and electronic coupling.^30^ These elements also exhibit relatively high *d*_c_ values, enhancing their ability to anchor reactive intermediates. This electronic heterogeneity, a hallmark of HEAs, enables site-specific tuning of adsorption energies, in line with the Sabatier principle.^23^ Altogether, this multidescriptor screening identifies Mo and W as the most promising dopants for FeCoNiCu, offering an optimal combination of structural stability and electronic properties for efficient HER catalysis. The following sections present the theoretical validation of HER pathways and the experimental realization of the selected HEA catalysts.

### 2.2 Unraveling the Alkaline HER Mechanism on HEA Surfaces

The alkaline HER process on the catalyst surface primarily proceeds via two main steps as depicted in Figure 2A: (i) the Volmer step, involving the dissociation of water (H_2_O → H* + OH), and (ii) the Heyrovsky (H + H_2_O + e^–^ → H_2_ + OH) or Tafel (2H → H_2_) step, leading to H_2_ formation.^31^ Due to the inherently weak adsorption and high energy barriers associated with breaking the H–OH bond, the Volmer step typically represents the rate-determining step (RDS) under alkaline conditions.^31^

**Figure 2:**
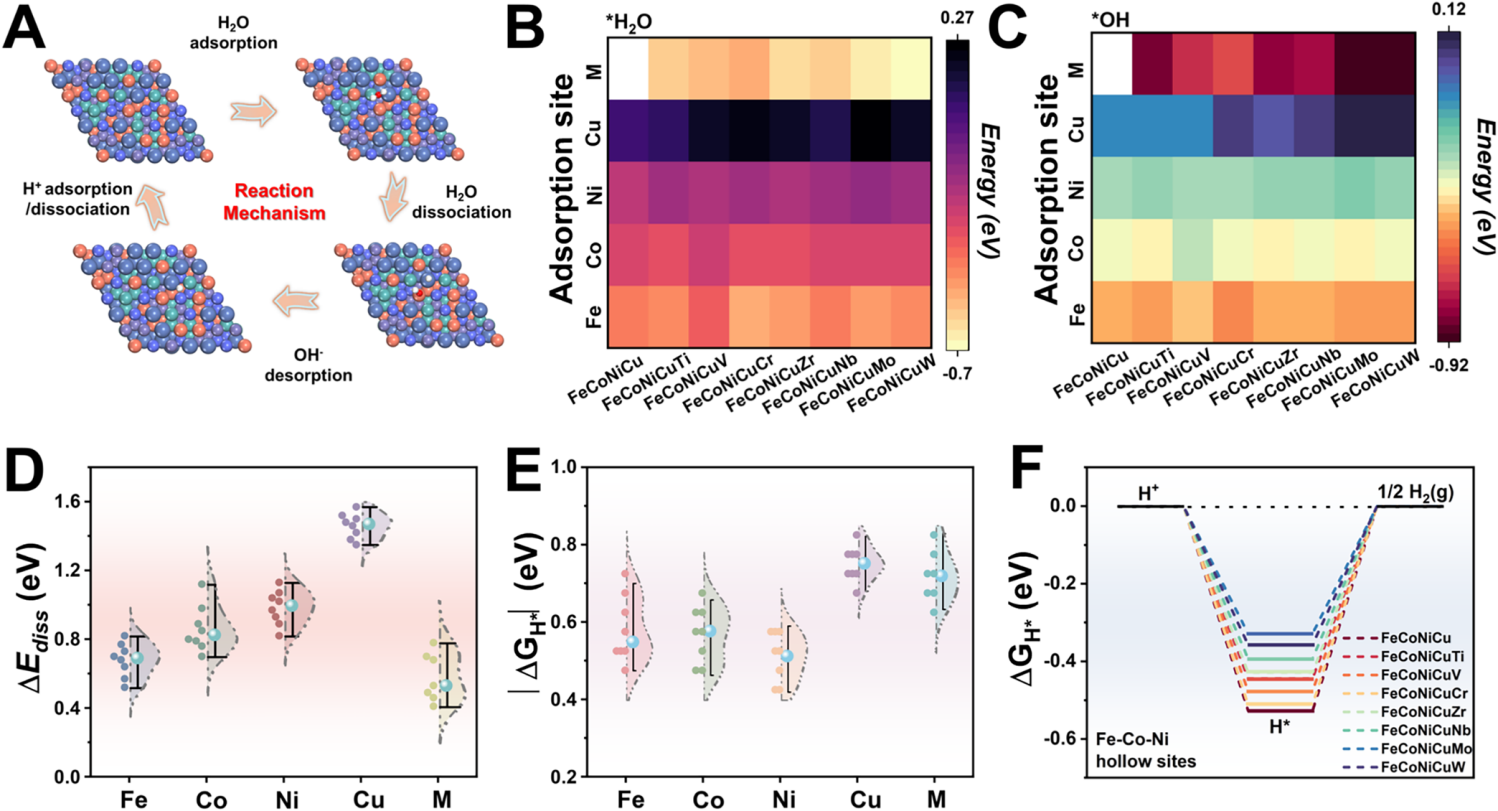
DFT analysis of alkaline HER pathways on FeCoNiCuM HEA surfaces. (A) Schematic illustration of the HER mechanism. (B) Calculated H_2_O adsorption energies and (C) OH^–^ adsorption energies at Fe, Co, Ni, Cu, and M top sites across various FeCoNiCuM HEA models. (D) Violin plots of calculated H_2_O dissociation energy barriers (Δ*E*_diss_), and (E) violin plots of hydrogen adsorption free energies (Δ*G*_H*_); median values are marked by blue dots; (F) Δ*G*_H*_ values calculated at Fe–Co–Ni hollow sites.

To elucidate how elemental composition influences the HER pathway, we systematically evaluated the adsorption behavior of H_2_O and *OH species across various active sites in the FeCoNiCuM HEA models (Figure 2B,C, Tables S8 and S9). Adsorption energies (*E*_ads_) were averaged over three representative top sites for each element to ensure robustness and statistical reliability. Stronger adsorption of H_2_O and *OH typically facilitates faster water dissociation by enhancing electron transfer and stabilizing key intermediates.^32,33^ As expected, oxophilic dopants such as Ti, Zr, V, Nb, Mo, and W exhibit significantly more negative *E*_ads_ values, indicative of strong interactions with oxygen-containing species. In contrast, base metal sites show a gradation of adsorption strength, with Fe exhibiting moderately strong binding, followed by Co and Ni, while Cu sites display the weakest, or even slightly positive, adsorption energies. This suggests that Fe plays a primary role in anchoring intermediates during the Volmer step, whereas Cu may promote efficient desorption and prevent surface poisoning, thereby complementing the overall reaction kinetics.^8^

These adsorption trends correlate directly with calculated water dissociation energy barriers (Δ*E*_diss_, Figures 2D, S20, and Table S10). Mo and W-doped alloys (FeCoNiCuMo and FeCoNiCuW) show significantly lower Δ*E*_diss_ values at Mo/W sites (0.41 eV for Mo, 0.46 eV for W) compared to the M sites in the same alloys (Figure 2D), whereas Cu sites exhibit higher barriers, attributed to weaker interactions and electron transfer characteristics. These findings suggest superior intrinsic water dissociation capabilities for HEAs incorporating Mo or W.

While strong *OH adsorption on oxophilic sites facilitates H_2_O dissociation, excessively strong binding can inhibit catalyst turnover by blocking active sites.^29^ Therefore, efficient OH desorption is essential for sustaining the HER activity. The compositional heterogeneity of HEAs offers a distinct advantage in this context, enabling the spatial separation of reaction functions: OH species formed on strongly adsorbing sites (e.g., Mo or W) can migrate to adjacent weakly adsorbing sites (e.g., Ni or Cu) for facile desorption. Our calculated desorption free energies (Δ*G*_OH*_), summarized in Table S11 and Figure S21, confirm that Cu sites, particularly in FeCoNiCuMo and FeCoNiCuW, exhibit the lowest OH desorption barriers, supporting their role as effective release sites. In contrast, fifth-element dopant sites (M) show substantially higher Δ*G*_OH*_ values, consistent with their strong oxophilicity. Importantly, a linear scaling relationship emerges between Δ*G*_OH*_ and Δ*E*_diss_ (Figure S21), suggesting that HEA surfaces can simultaneously enable efficient water dissociation on M sites and rapid *OH clearance via Cu sites.^34^ Fe, Co, and Ni, with intermediate adsorption and desorption energetics, likely serve as versatile sites that bridge these processes and facilitate the subsequent hydrogen evolution steps.

Following water dissociation, the adsorption free energy of hydrogen (Δ*G*_H*_) serves as a critical descriptor of HER performance.^35^ For optimal catalytic activity, Δ*G*_H*_ should be close to zero, indicating a balance between sufficient H* adsorption and facile H_2_ desorption. As shown in Figures 2E, S22, and Table S12, most metal sites in the FeCoNiCuM HEAs exhibit exothermic H* adsorption, with Δ*G*_H*_ < 0. Among these, Fe, Co, and Ni sites demonstrate the most favorable binding characteristics,^36^ with median Δ*G*_H*_ values following the trend: Ni > Co ≈ Fe > M > Cu. This order correlates with the previously discussed d-band center distributions (Figure S19B), where moderately elevated d-band centers on Fe, Co, and Ni align with the Sabatier principle, favoring neither too strong nor too weak H* adsorption. Further insights are provided by crystal orbital Hamilton population (COHP) analysis (Figure S23), which reveals a volcano-type relationship between the integral COHP (ICOHP) values and Δ*G*_H*_. FeCoNiCuMo and FeCoNiCuW are positioned near the volcano apex, indicating optimal M–H bonding strength for catalytic activity.^37^ Notably, when evaluating hydrogen adsorption on Fe–Co–Ni hollow sites (Figure 2F), FeCoNiCuMo exhibits a Δ*G*_H*_ value closest to the thermoneutral ideal (∼0 eV), suggesting particularly favorable H* adsorption/desorption kinetics at these multimetallic ensemble sites.^38^ This is further supported by 2D differential charge density maps (Figure S24), which show localized electron accumulation and depletion around the adsorbed H*, indicative of stable yet reversible bonding. Together, these findings reinforce FeCoNiCuMo as a leading candidate with finely tuned hydrogen adsorption energetics.

Taken together, our comprehensive theoretical analysis identifies FeCoNiCuMo as the most promising HER catalyst among the evaluated quinary HEAs. This composition exhibits consistently favorable energetics across all key mechanistic descriptors including water adsorption and dissociation, OH desorption, and H binding. Based on these insights, FeCoNiCuMo, alongside FeCoNiCuW, FeCoNiCuV, and the pristine FeCoNiCu alloy, was selected for experimental validation to corroborate the predictions of our data-driven computational framework.

### 2.3 Synthesis and Characterization of FeCoNiCuM HEA Films

Guided by the computational screening results, a series of HEA films, namely, the quaternary FeCoNiCu and the quinary FeCoNiCuV, FeCoNiCuMo, and FeCoNiCuW, were synthesized via magnetron sputtering (workflow illustrated in Figure 1A). The films were deposited onto nickel foam (NF) substrates for electrochemical testing and onto carbon paper (CP) for structural and compositional characterization. XRD patterns of the CP-supported films (Figure 3A) reveal that all compositions adopt a face-centered cubic (fcc) crystal structure, as indicated by the characteristic diffraction peaks at ∼43 and ∼51°, corresponding to the (111) and (200) planes,^39^ respectively. Among them, the FeCoNiCuMo film displays broader and less intense peaks, suggesting reduced crystallite size, increased lattice strain, structural defects, or film–substrate interfacial effects.^9^ To confirm the phase purity, synchrotron XRD was performed on the FeCoNiCuMo film (Figure 3B). The resulting pattern shows sharp, well-defined reflections assigned exclusively to fcc lattice planes—(111), (200), (220), (311), and (222)—with no detectable secondary phases or intermetallics.^40^ This unambiguous identification of a single-phase fcc structure is essential for confirming the HEA nature of the synthesized material.

**Figure 3:**
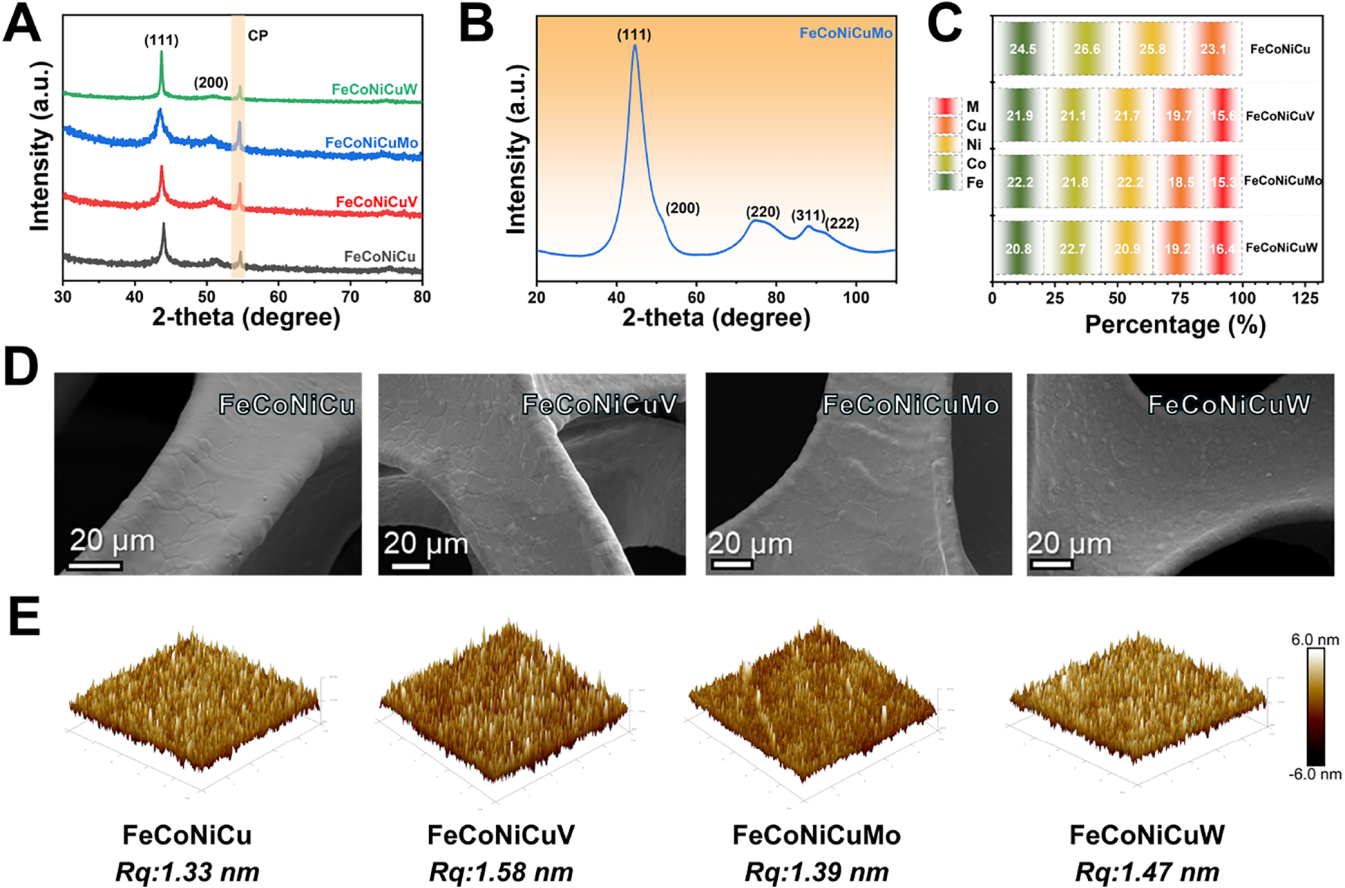
Structural, compositional, and morphological characterization of sputtered FeCoNiCuM HEA films. (A) XRD patterns of FeCoNiCu, FeCoNiCuV, FeCoNiCuMo, and FeCoNiCuW films deposited on carbon paper. (B) Synchrotron XRD pattern of the FeCoNiCuMo film. (C) Elemental compositions (atom %) determined by EDS. (D) SEM images of the film surfaces. (E) AFM images and corresponding root-mean-square roughness (*R*_q_) values.

The elemental compositions of the synthesized HEA films were determined by energy-dispersive X-ray spectroscopy (EDS), and the results are summarized in Figure 3C. The quaternary FeCoNiCu films exhibit near-equiatomic distributions of the base metals, confirming uniform elemental mixing. In the quinary alloys, the fifth elements, V, Mo, or W, were successfully incorporated at atomic concentrations of approximately 15–16 atom %, demonstrating precise compositional control during the sputtering process. These results validate the intended doping strategy and confirm the homogeneity of the FeCoNiCuM HEA films. While more precise techniques such as ICP or XRF can be employed for elemental quantification, EDS is a standard and widely accepted method for thin-film HEAs and, in our case, reliably confirms both elemental incorporation and compositional homogeneity, in good agreement with the supplementary XPS analysis (discussed later in Figure S27D).

SEM images (Figure 3D) reveal that all as-deposited HEA films form dense, continuous, and uniform coatings across the substrate surfaces. Cross-sectional SEM analysis (Figure S25) confirms a consistent film thickness of approximately 500 nm, underscoring the reliability and reproducibility of the magnetron sputtering process for fabricating homogeneous HEA thin films. Atomic force microscopy (AFM) measurements (Figure 3E) were employed to quantify the surface topography and roughness. The quaternary FeCoNiCu film exhibits an average roughness (*R*_q_) of 1.33 nm, while the quinary variants display slightly increased values: FeCoNiCuV (1.58 nm), FeCoNiCuMo (1.39 nm), and FeCoNiCuW (1.47 nm). Given the identical deposition parameters, this modest roughening is likely driven by changes in microstructure and surface growth kinetics induced by the incorporation of the fifth element.^41^ These surface variations may influence the electrochemically active surface area and local mass transport properties, thereby affecting the catalytic performance.

Figure 4A–D presents TEM, SAED, and HRTEM images for the FeCoNiCu, FeCoNiCuV, FeCoNiCuMo, and FeCoNiCuW films. The TEM images reveal that the films, when mechanically delaminated, consist of agglomerated nanoparticles. The corresponding SAED patterns for all compositions exhibit continuous and well-defined diffraction rings, which can be indexed to the (111), (200), (220), and (311) planes of a fcc lattice.^42^ These observations confirm the polycrystalline nature of the films and are in excellent agreement with the XRD findings. HRTEM images further reveal distinct lattice fringes in each sample. The measured interplanar spacings associated with the {111} planes are approximately 0.208 nm for FeCoNiCu, 0.206 nm for FeCoNiCuV, 0.209 nm for FeCoNiCuMo, and 0.208 nm for FeCoNiCuW, values that are consistent with those expected for fcc FeCoNiCu-based alloys and in line with literature reports.^9,21,42^ The slight variations in *d*-spacing across the different HEA compositions reflect local lattice distortions, which are hallmarks of high-entropy systems. These distortions originate from the random incorporation of elements with differing atomic radii, particularly V, Mo, and W, within the single-phase solid solution matrix (see Table S1).

**Figure 4:**
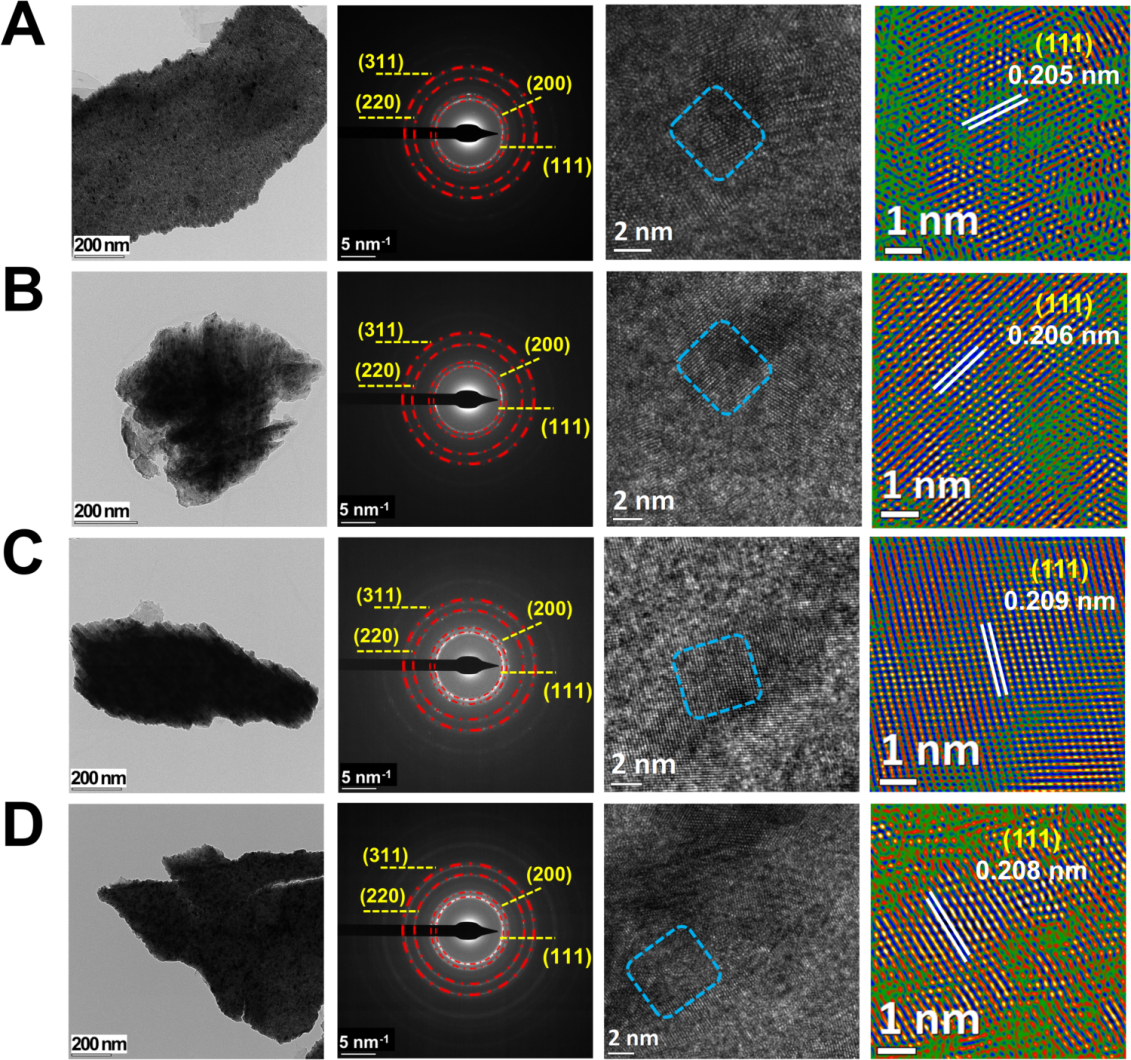
TEM analysis of (A) FeCoNiCu, (B) FeCoNiCuV, (C) FeCoNiCuMo, and (D) FeCoNiCuW films. For each sample, images are presented sequentially from left to right: TEM image, SAED pattern, HRTEM image, and corresponding interplanar spacing measurements associated with the {111} planes.

To assess nanoscale chemical uniformity, high-angle annular dark-field scanning transmission electron microscopy (HAADF-STEM) combined with EDS mapping was conducted (Figure S26). The EDS elemental maps for FeCoNiCu, FeCoNiCuV, FeCoNiCuMo, and FeCoNiCuW reveal a homogeneous spatial distribution of all constituent elements, including Fe, Co, Ni, and Cu and the respective fifth element (V, Mo, or W), across the examined regions. The absence of detectable elemental segregation confirms the formation of a chemically disordered solid solution, consistent with the HEA design. These results provide strong nanoscale evidence supporting the structural and compositional stability of the synthesized HEA films. This homogeneous single-phase nature rules out the possibility of a simple physical mixture of quaternary alloys and single-metal oxides, underscoring that the observed catalytic performance arises from the synergistic interactions intrinsic to the HEA structure. In summary, the combined structural and compositional analyses—spanning XRD, TEM, SAED, HRTEM, and HAADF-STEM with EDS mapping, confirm that magnetron sputtering yields well-crystallized, single-phase fcc HEA films (FeCoNiCu, FeCoNiCuV, FeCoNiCuMo, and FeCoNiCuW) with uniform elemental distribution and no evidence of phase separation or segregation.

High-resolution X-ray photoelectron spectroscopy (XPS) was employed to probe the surface chemical states of the constituent elements. The Fe 2p, Co 2p, Ni 2p, and Cu 2p core-level spectra are presented in Figure 5A–D, while the successful incorporation of V, Mo, and W is confirmed by their characteristic V 2p, Mo 3d, and W 4f signals, respectively (Figure S27). Notably, the high-resolution V 2p, Mo 3d, and W 4f spectra reveal predominant metallic features, accompanied by inevitable surface oxidation states (e.g., V^0^/V^4+^, Mo^0^/Mo^4+^/Mo^6+^, and W^0^/W^4+^/W^6+^) arising from their intrinsic redox potentials. The coexistence of metallic and oxidized species indicates that, while surface oxidation is unavoidable under ambient exposure, the dominant metallic contributions confirm the successful incorporation of these elements into the HEA lattice. Such mixed surface states are expected to influence both the electronic interactions within the alloy and its catalytic stability under alkaline HER conditions. The Fe 2p spectra (Figure 5A) exhibit complex multiplet features attributed to metallic Fe^0^ and oxidized Fe species (Fe^2+^ and Fe^3+^),^43^ along with satellite peaks. The prominence of oxidized iron is expected, given Fe’s low redox potential and high susceptibility to ambient oxidation (Table S13).^8^ In contrast, the Co 2p (Figure 5B) and Ni 2p (Figure 5C) spectra are dominated by signals corresponding to metallic Co^0^ and Ni^0^, with minor contributions from Co^2+^ and Ni^2+^, as evidenced by weaker satellite features. The Cu 2p spectrum (Figure 5D) is similarly dominated by peaks associated with Cu^0^, with minimal evidence of oxidation, indicating greater resistance to surface oxidation for Cu, Ni, and Co relative to Fe.^9^

**Figure 5:**
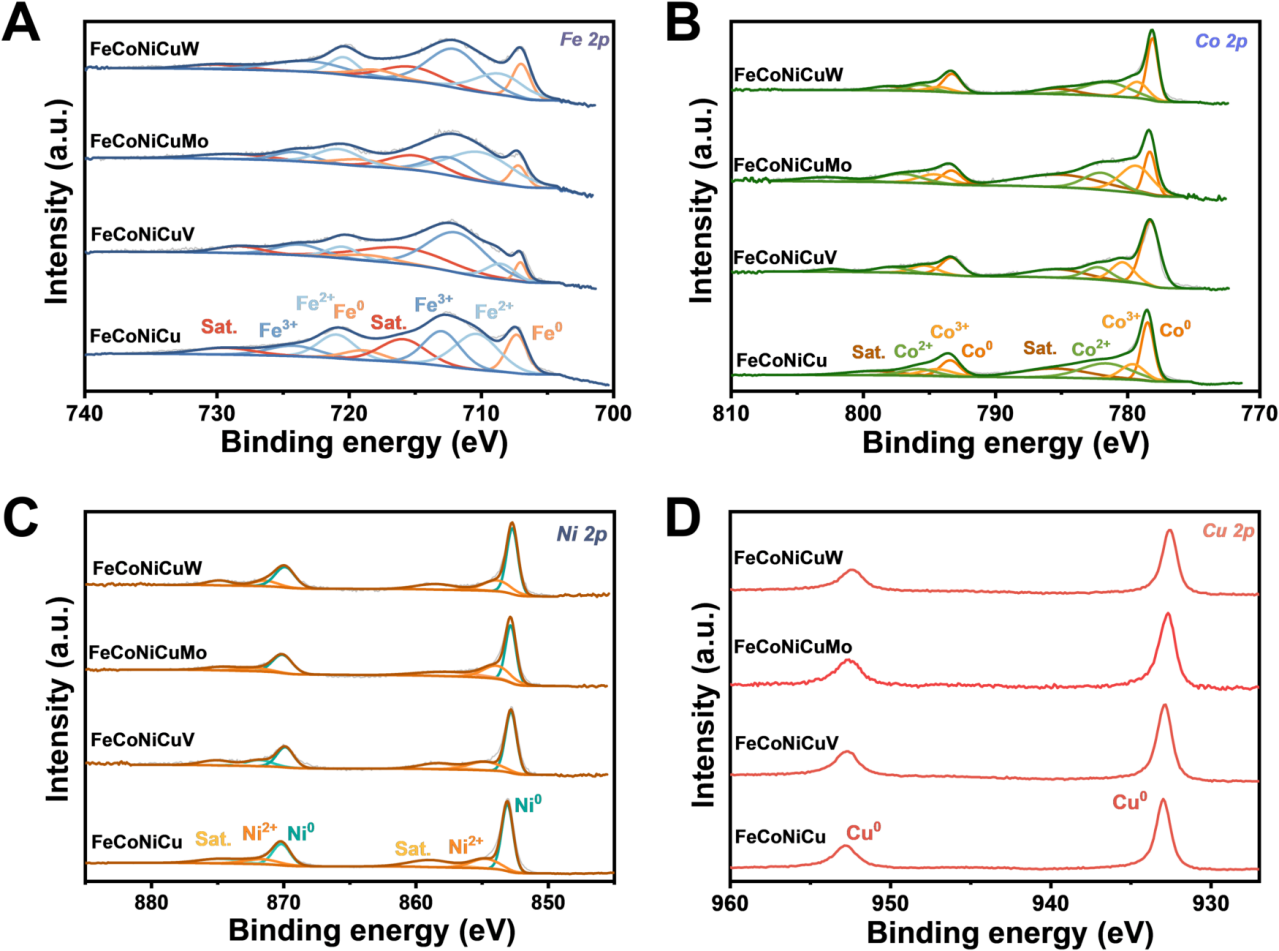
Surface chemical state analysis of FeCoNiCuM HEA films by XPS. High-resolution XPS spectra of the (A) Fe 2p, (B) Co 2p, (C) Ni 2p, and (D) Cu 2p core levels for the FeCoNiCu, FeCoNiCuV, FeCoNiCuMo, and FeCoNiCuW films.

A key observation across all core-level spectra is the systematic shift in binding energies for the quinary HEA films relative to the quaternary FeCoNiCu alloy and pure elemental standards (Figure S27E,F). These shifts reflect changes in the local electronic environment due to alloying, indicative of charge redistribution and strong electronic interactions among the constituent elements. This observation is consistent with theoretical predictions of modified electronic structures in HEAs and further underscores the robust interelement hybridization characteristic of chemically disordered, multicomponent systems. Such electronic perturbations are expected to play a crucial role in tuning the catalytic activity of HEA surfaces.

### 2.4 Electrocatalytic Performance for Alkaline HER

The alkaline HER performance of the synthesized HEA films, FeCoNiCu and FeCoNiCuM (*M* = V, Mo, W), was systematically evaluated in 1.0 M KOH by using a standard three-electrode configuration. For benchmarking, a commercial Pt/C catalyst supported on NF was tested under identical conditions. Linear sweep voltammetry (LSV) curves recorded at a scan rate of 5 mV s^–1^ are shown in Figure 6A. Among the HEA films, FeCoNiCuMo exhibited the highest catalytic activity, outperforming both FeCoNiCuV and FeCoNiCuW, as well as the base quaternary FeCoNiCu alloy. Overpotentials required to achieve a current density of 10 mA cm^–2^ (η_10_) are compared in Figure 6B. FeCoNiCuMo reached η_10_ at just 60.1 mV. This was significantly lower than those of FeCoNiCuW (91.2 mV), FeCoNiCuV (107.9 mV), and FeCoNiCu (114.3 mV) and approached the performance of the Pt/C benchmark (39.9 mV). Additionally, FeCoNiCuMo displayed a more rapid increase in current density with applied potential, reflecting intrinsically favorable reaction kinetics and superior HER catalytic behavior.

**Figure 6:**
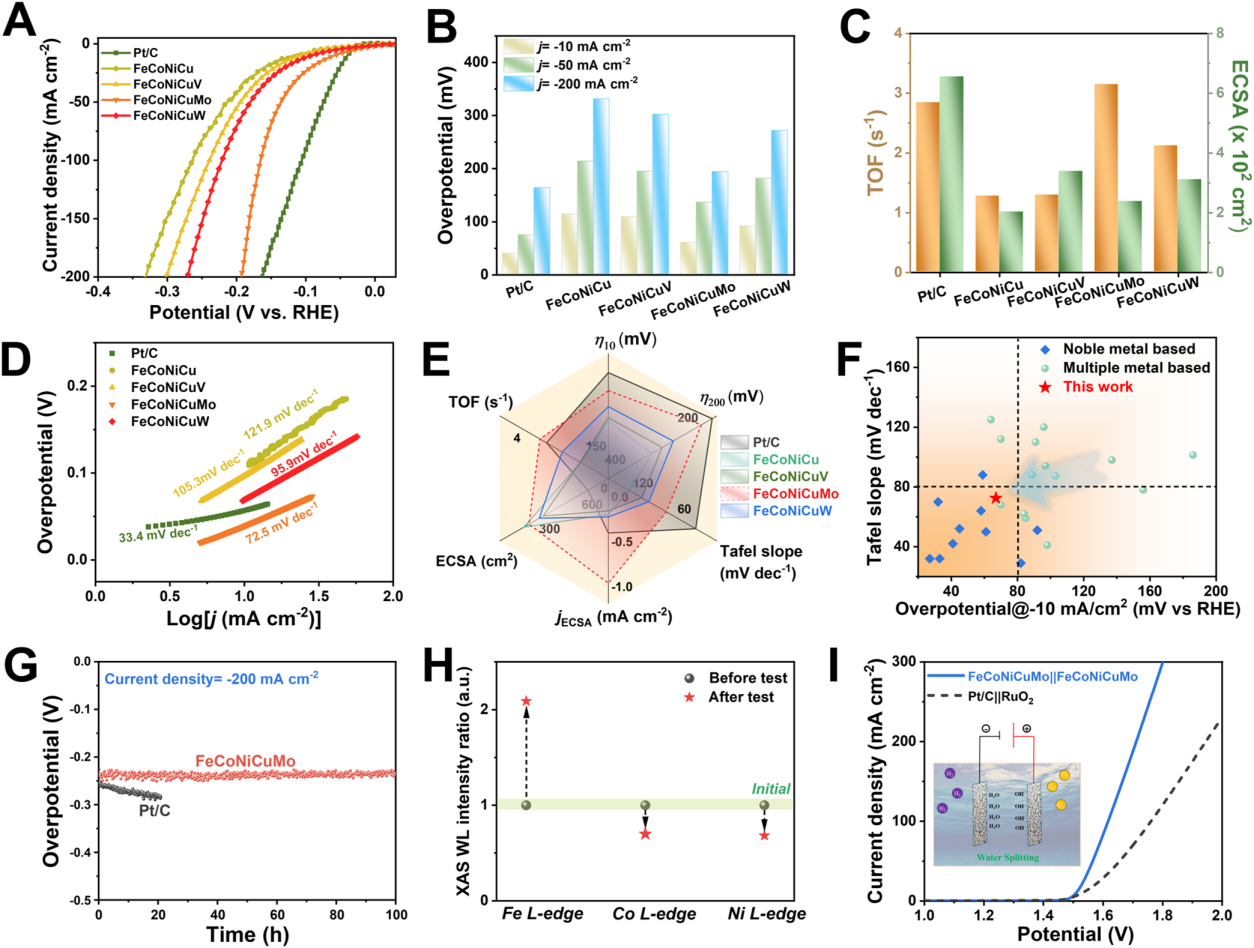
Electrocatalytic performance and stability of FeCoNiCuM HEA films for alkaline HER and overall water splitting in 1.0 M KOH. (A) Linear sweep voltammetry (LSV) curves. (B) Overpotentials required to reach current densities of 10, 50, and 200 mA cm^–2^ for each catalyst. (C) TOF values at a fixed overpotential and ECSA derived from double-layer capacitance (*C*_dl_) measurements. (D) Tafel plots extracted from the LSV data, reflecting HER kinetics. (E) Radar chart summarizing key HER performance metrics. (F) Benchmarking of FeCoNiCuMo against state-of-the-art alkaline HER catalysts based on overpotential (η_10_) and Tafel slope. (G) Chronopotentiometric stability test of FeCoNiCuMo and commercial Pt/C catalyst at −200 mA cm^–2^ for 100 h. (H) Synchrotron sXAS analysis of Fe, Co, and Ni L-edge white-line (WL) intensities before and after HER durability testing. (I) Two-electrode overall water-splitting performance: polarization curves comparing FeCoNiCuMo∥FeCoNiCuMo and Pt/C∥RuO_2_ electrolyzer configurations.

To evaluate the intrinsic catalytic activity independent of geometric effects, the LSV curves were normalized to the electrochemically active surface area (ECSA). ECSA values were derived from double-layer capacitance (*C*_dl_) measurements (Figure S28),^44^ and the corresponding ECSA-normalized LSV curves are shown in Figure S29. Among the HEA catalysts, FeCoNiCuMo exhibited the highest specific activity, closely approaching that of the Pt/C benchmark. This superior intrinsic performance is further validated by turnover frequency (TOF) calculations (Figure 6C), where FeCoNiCuMo unexpectedly surpasses Pt/C at comparable overpotentials. Notably, this exceptional activity is achieved using only earth-abundant elements, highlighting the effectiveness of the data-driven computational screening in identifying high-performance HER catalysts for alkaline media.

Further insights into the HER kinetics were obtained through Tafel analysis. As shown in Figure 6D, the FeCoNiCuMo catalyst exhibits a Tafel slope of 72.5 mV dec^–1^, significantly lower than those of FeCoNiCuW (85.9 mV dec^–1^), FeCoNiCuV (105.3 mV dec^–1^), and the base FeCoNiCu alloy (121.9 mV dec^–1^), indicating markedly improved HER kinetics upon Mo incorporation. This value suggests that the hydrogen evolution on FeCoNiCuMo proceeds via a Volmer–Heyrovsky mechanism,^8^ with electrochemical desorption serving as the rate-determining step. Complementary electrochemical impedance spectroscopy (EIS) measurements (Figure S30) reveal a notably smaller charge transfer resistance (*R*_ct_) for FeCoNiCuMo relative to the other HEAs, further confirming more efficient interfacial charge transport.^45^ A comprehensive comparison of key performance indicators—including η_10_, η_200_ (overpotential at 200 mA cm^–2^), Tafel slope, ECSA-normalized specific activity, TOF, and ECSA, is provided in the radar chart (Figure 6E), which clearly underscores the superior catalytic profile of FeCoNiCuMo. Moreover, when benchmarked against a range of recently reported noble-metal- and multimetal-based HER catalysts in alkaline media (Figure 6F, Tables S14 and S15), FeCoNiCuMo exhibits highly competitive electrocatalytic performance, despite being composed entirely of earth-abundant elements.

The long-term operational stability of the FeCoNiCuMo electrocatalyst and commercial Pt/C catalysts was assessed via chronopotentiometry at a high current density of −200 mA cm^–2^ in 1.0 M KOH for 100 h. As shown in Figure 6G, the overpotential for FeCoNiCuMo required to sustain this current remained remarkably stable over the entire testing period. These improved stabilities against Pt/C, tested under identical conditions, further highlight the outstanding durability of FeCoNiCuMo and establish its clear advantage over both commercial noble-metal benchmarks and the quaternary alloy references presented in Figure 6A. Poststability structural and compositional analyses were conducted using TEM and EDS (Figure S31). These characterizations confirmed that the FeCoNiCuMo film retained its structural integrity, with no observable surface degradation, morphological changes, or elemental leaching after prolonged HER operation. This outstanding stability underscores the robustness of the HEA architecture and further validates the effectiveness of Mo incorporation in enhancing both activity and durability.

To probe the evolution of the electronic structure during catalysis, synchrotron-based soft X-ray absorption spectroscopy (sXAS) was performed on the FeCoNiCuMo film before and after the HER durability test (Figure S32). As shown in Figure 6H, a pronounced increase in the white-line (WL) intensity at the Fe L-edge was observed postoperation, while the WL intensities at the Co and Ni L-edges exhibited a corresponding decrease. This trend suggests dynamic electron redistribution within the HEA matrix,^46^ with Fe sites donating electron density to Co and Ni sites during HER. Such an electron transfer pathway may be facilitated by the presence of Mo 4d orbitals, which likely contribute to stabilizing the evolving electronic environment under reaction conditions.^47^ While additional sXAS analysis of Cu and Mo would be informative, our combined DFT (Figures S18 and S19), XPS (Figure S27), and spectroscopic evidence already capture their roles, and the Fe, Co, and Ni L-edge data were specifically chosen to probe the Mo-mediated electronic redistribution within the 3d-metal matrix. This cooperative electronic behavior implies a synergistic role distribution, wherein Fe sites preferentially mediate the Volmer step (H_2_O dissociation) while Co and Ni sites optimize hydrogen adsorption and evolution. These findings not only align with theoretical electronic structure predictions but also reinforce the validity of our data-driven HEA design strategy.

Building on the exceptional HER performance of FeCoNiCuMo, its catalytic activity toward the oxygen evolution reaction (OER) was also investigated to evaluate its viability as a bifunctional electrocatalyst for overall water splitting. As shown in Figure S33, the FeCoNiCuMo HEA film demonstrates notable OER activity in 1.0 M KOH, with performance metrics—such as the overpotential at 10 mA cm^–2^ (η_10_), Tafel slope, charge transfer resistance, and stability—comparable to or surpassing those of the commercial RuO_2_ benchmark. Leveraging its bifunctional nature, an alkaline electrolyzer was assembled using FeCoNiCuMo as both the anode and the cathode (FeCoNiCuMo||FeCoNiCuMo). For benchmarking, a conventional Pt/C||RuO_2_ electrolyzer was also evaluated. As depicted in Figure 6I, the FeCoNiCuMo-based two-electrode system delivers superior overall water-splitting performance, achieving high current densities at lower cell voltages compared to the Pt/C||RuO_2_ couple. These results underscore the remarkable bifunctionality, durability, and cost-effectiveness of FeCoNiCuMo, positioning it as a promising candidate for practical alkaline water electrolysis applications.

## 3 Conclusions

In summary, this study demonstrates a robust, descriptor-guided, data-driven strategy for the rational design and experimental realization of high-performance FeCoNiCu-based HEA electrocatalysts for alkaline hydrogen evolution. By moving beyond single-descriptor approaches, our multiparameter computational framework, encompassing adsorption energetics, d-band center analysis, and kinetic descriptors, efficiently navigated the complex compositional space, identifying Mo and W as optimal dopants for enhancing both structural stability and catalytic functionality.

The FeCoNiCuMo HEA film, synthesized via magnetron sputtering, validated these predictions experimentally. It exhibited outstanding HER activity in 1.0 M KOH, with a low overpotential of 60.1 mV at 10 mA cm^–2^ and excellent durability over 100 h at −200 mA cm^–2^. Its performance not only exceeded that of the base FeCoNiCu alloy and other quinary variants but also rivaled those of commercial noble-metal benchmarks. Operando and ex situ spectroscopic analyses, supported by theoretical calculations, revealed that Mo incorporation modulates the local electronic environment, enabling synergistic site-specific activity for water dissociation and hydrogen evolution.

Beyond delivering a cost-effective and earth-abundant catalyst, this work underscores the power of integrating high-throughput computational screening with targeted experimental validation to accelerate the discovery of complex multielement materials. The design principles and integrated workflow established here offer a transferable blueprint for the development of next-generation electrocatalysts with tailored functionalities. This strategy, grounded in mechanistic insight, holds broad applicability for electrochemical energy conversion and storage technologies, including overall water splitting, CO_2_ reduction, and nitrogen fixation, ultimately advancing the development of more efficient and sustainable catalytic systems.

## 4 Experimental Section

### 4.1 Chemicals

High-purity metal targets were obtained from Zhongsheng Hengan (Beijing) New Material Technology Co., Ltd. Ruthenium oxide (RuO_2_, 99.95%, Sigma-Aldrich), commercial 20% Pt/C (Sigma-Aldrich), and potassium hydroxide (KOH, 99.99%, Sigma-Aldrich) were used without further purification. All aqueous solutions were prepared using deionized water (18.2 MΩ cm) obtained from an ultrapure purification system.

### 4.2 Deposition of FeCoNiCu and FeCoNiCuM High-Entropy Alloy Films

High-entropy alloy (HEA) thin films with nominal compositions of FeCoNiCu, FeCoNiCuV, FeCoNiCuMo, and FeCoNiCuW were fabricated on carbon paper and nickel foam substrates via pulsed DC reactive magnetron sputtering.^48^ The deposition employed composite Fe/Co/Ni/Cu/V/Mo/W targets at a constant power of 100 W. A pulsed DC power source operating at a frequency of 100 kHz with a pulse-on duration of 200 μs and an 80% duty cycle was utilized to ensure stable plasma conditions during film growth. Prior to deposition, all targets were pretreated by Ar^+^ ion bombardment for 10 min to eliminate surface oxides and residual contaminants, thus ensuring clean surface conditions. The vacuum chamber was evacuated to a base pressure of 5.0 × 10^–4^ Pa, and the working pressure was maintained at 0.5 Pa throughout the sputtering process. High-purity Ar gas (99.99%) served as the sputtering medium, introduced at a controlled flow rate of 30 sccm. The target-to-substrate distance was fixed at 150 mm to promote a uniform film deposition.

### 4.3 Synthesis of Pt/C and RuO_2_ Electrodes

Commercial Pt/C and RuO_2_ powders were employed as benchmark electrocatalysts for the hydrogen evolution reaction and oxygen evolution reaction, respectively, and were deposited onto nickel foam substrates via a drop-casting method. Specifically, 5 mg of either Pt/C or RuO_2_ catalyst was ultrasonically dispersed for 1 h in a mixed solvent composed of 500 μL of 2-propanol, 480 μL of deionized water, and 20 μL of 5 wt % Nafion solution (DuPont) to ensure uniform dispersion. Subsequently, 80 μL of the homogeneous catalyst ink was drop-cast onto a precut NF electrode (approximately 1 × 1 cm^2^), resulting in a catalyst loading of ∼0.40 mg cm^–2^. The electrodes were then dried under ambient conditions to facilitate solvent evaporation and ensure stable catalyst adhesion.

### 4.4 Characterizations

The microstructural and compositional characteristics of the samples were comprehensively examined by using multiple advanced characterization techniques. Field-emission scanning electron microscopy (FESEM, FEI Sirion, operated at 20 kV) coupled with energy-dispersive X-ray spectroscopy was employed to assess the surface morphology and elemental distribution. Surface topography and roughness were further quantified by atomic force microscopy (AFM, Bruker Dimension ICON) within a scanning area of 5 × 5 μm^2^. High-angle annular dark-field scanning transmission electron microscopy was performed using a Talos F200X microscope equipped with an EDS detector to obtain high-resolution microstructural and compositional information.

Crystallographic features were investigated via X-ray diffraction using a Bruker D8 diffractometer operated at 40 kV and 35 mA with monochromatic Cu Kα radiation. To complement lab-based XRD, synchrotron X-ray diffraction measurements were conducted at the Material Science beamline of the SESAME synchrotron facility in Jordan,^49,50^ using 15 keV incident X-rays (λ = 0.8272 Å). The SXRD setup incorporated a double-crystal Si (111) Kohzu monochromator with a sagittal focus on the second crystal and two Rh-coated mirrors to optimize beam collimation and purity in the wiggler-based beamline environment.

The chemical states of constituent elements were probed by X-ray photoelectron spectroscopy (XPS, Thermo Fisher ESCALAB Xi+), utilizing monochromatic Al Kα radiation. All binding energies were calibrated against the C 1s peak at 284.6 eV from adventitious carbon. Soft X-ray absorption spectroscopy (XAS) was further performed at the Fe L-edge, Co L-edge, and Ni L-edge in partial fluorescence yield (PFY) mode at the PIRX beamline of the SOLARIS National Synchrotron Radiation Centre (Poland).

### 4.5 Electrochemical Measurements

All electrochemical measurements were conducted at ambient temperature using a CHI 660E electrochemical workstation in a conventional three-electrode configuration. The HEA films deposited on nickel foam with a geometric area of 1 × 1 cm^2^ served as the working electrodes. A standard Hg/HgO electrode was employed as the reference electrode (*E*_RHE_ = *E*_Hg/Hgo_ + 0.0591 × pH + 0.098), while a graphite rod was used as the counter electrode. Unless otherwise specified, all electrochemical potentials were corrected for the *iR* drop using an 85% compensation ratio.

Hydrogen evolution reaction (HER) measurements were carried out in a 1.0 M KOH aqueous electrolyte. Linear sweep voltammetry (LSV) was performed at a scan rate of 5 mV s^–1^. Electrochemical impedance spectroscopy (EIS) was recorded at −0.05 V vs RHE over a frequency range of 0.01 Hz to 10^5^ Hz, with a perturbation amplitude of 10 mV. The electrochemically active surface area was estimated via the double-layer capacitance, determined from cyclic voltammetry curves collected at scan rates of 20 to 100 mV s^–1^ within the non-Faradaic region. A specific capacitance value of 40 μF cm^–2^ was employed for ECSA calculation.^6^ Chronopotentiometry tests for HER durability were conducted at a constant current density of −200 mA cm^–2^ for 100 h.

Oxygen evolution reaction (OER) measurements were also performed in 1.0 M KOH under identical conditions. LSV and Tafel analyses were conducted at a scan rate of 5 mV s^–1^. EIS measurements were collected at 1.5 V vs RHE using the same frequency range and amplitude settings. Long-term OER stability was evaluated via chronopotentiometry at 300 mA cm^–2^ for 100 h.

To assess overall water-splitting performance, a two-electrode electrolyzer was assembled using identical FeCoNiCuMo/NF electrodes (1 × 1 cm^2^) as both the anode and cathode.

### 4.6 Theoretical Calculations

First-principles calculations based on density functional theory (DFT) were carried out using the Cambridge Sequential Total Energy Package (CASTEP) module implemented in the Materials Studio software suite.^51^ The exchange–correlation interactions were treated within the generalized gradient approximation (GGA) using the Perdew–Burke–Ernzerhof (PBE) functional.^52^ Core–valence electron interactions were described by using the on-the-fly generated (OTFG) ultrasoft pseudopotentials. A plane-wave energy cutoff of 400 eV was applied for all calculations. To account for van der Waals (vdW) interactions, Grimme’s D2 dispersion correction was incorporated. Structural relaxations were performed using the limited-memory Broyden–Fletcher–Goldfarb–Shanno (LBFGS) algorithm with a medium-density Monkhorst–Pack k-point grid. The convergence thresholds for geometry optimization were set to 5 × 10^–5^ eV per atom for total energy, 0.001 eV Å^–1^ for maximum force, and 0.005 Å for maximum displacement. A vacuum layer of 20 Å was applied along the *z*-axis to eliminate interlayer interactions under periodic boundary conditions.

Molecular dynamics (MD) simulations were performed in the canonical (NVT) ensemble at a temperature of 350 K and an ambient pressure. A time step of 1 fs was adopted, with a total simulation duration of 5 ps, corresponding to 5000 integration steps. The mean square displacement (MSD) of each constituent element was evaluated as a function of time and calculated by averaging the squared atomic displacements across the simulation trajectory.

## Supplementary Material



## Notes

Additional characterization results (EDS mappings, SEM, TEM, XPS, XAS spectrum); electrocatalytic data (LSVs, EIS data, and comparison table); and theoretical simulations results (PDF)
