# Supplementary material for: Descriptor-Guided Design of Mo-Doped FeCoNiCu High-Entropy Alloy Electrocatalysts Surpassing Pt for Alkaline Hydrogen Evolution
Source: ACS Appl Mater Interfaces. 2025 Sep 11;17(38):53587–99. doi: 10.1021/acsami.5c13488 (PMC12464898; doi:10.1021/acsami.5c13488)
Supplement: Supplementary file 1 [file am5c13488_si_001.pdf]

Supporting Information for

Descriptor-Guided Design of Mo-Doped FeCoNiCu High-Entropy Alloy Electrocatalysts Surpassing Pt for Alkaline Hydrogen Evolution

Shiqi Wang [a,b,*], Haixian Yan [a], Wenyi Huo [c,d,*], Mahmoud Abdellatief [e], Feng Fang [a,*], and Pedro H.C. Camargo [b,*]

[a] *Jiangsu Key Laboratory of Advanced Metallic Materials, Southeast University, Nanjing 211189, P. R. China.*

[b] *Department of Chemistry, University of Helsinki, A.I. Virtasen aukio 1, PO Box 55, Helsinki FIN–0014, Finland.*

[c] *College of Mechanical and Electrical Engineering, Nanjing Forestry University, Nanjing 210037, P. R. China.*

[d] *NOMATEN Centre of Excellence, National Centre for Nuclear Research, Otwock 05-400, Poland.*

[e] *Synchrotron-light for Experimental Science and Applications in the Middle East (SESAME), Allan 19252, Jordan.*

** Corresponding authors:*

Email: pedro.camargo@helsinki.fi, fangfeng@seu.edu.cn, wyhuo@njfu.edu.cn, shiqi.z.wang@helsinki.fi

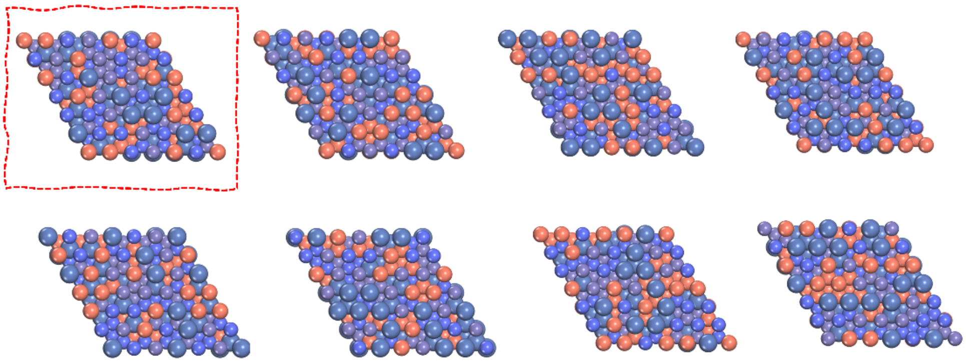

FeCoNiCu

Figure S1. Surface models of randomly generated FeCoNiCu configurations. The structure highlighted with a red dashed box corresponds to the configuration exhibiting the lowest surface energy.

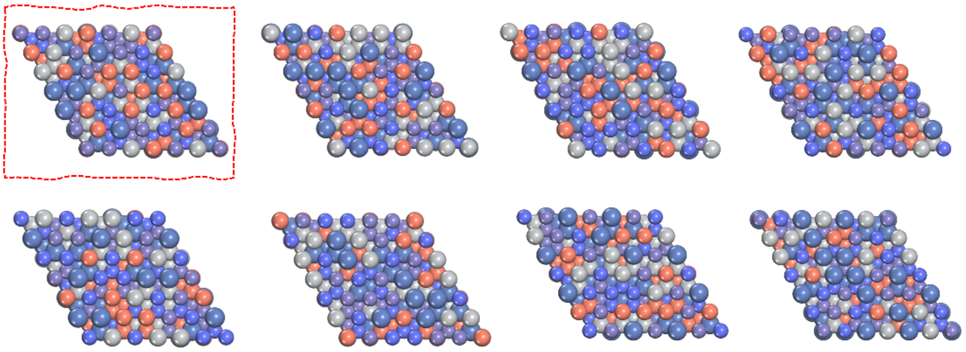

FeCoNiCuTi

Figure S2. Surface models of FeCoNiCuTi configurations. The red dashed box marks the model with the lowest surface energy among the randomly built structures.

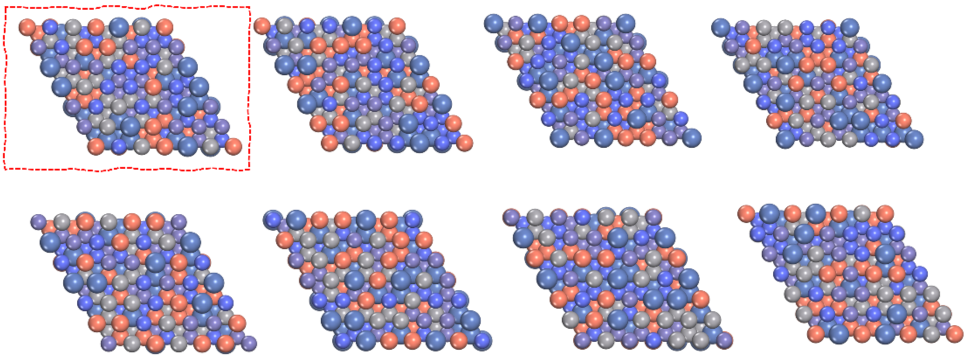

FeCoNiCuV

Figure S3. Randomly generated surface configurations of FeCoNiCuV. The structure with the lowest surface energy is highlighted by a red dashed box.

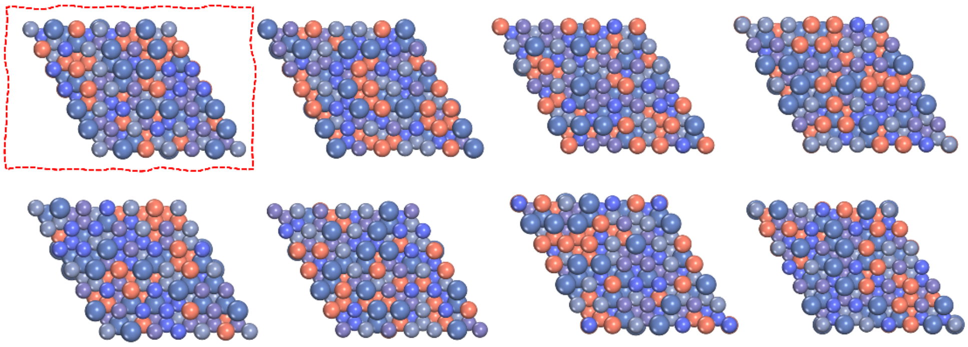

FeCoNiCuCr

Figure S4. Randomly generated surface models of FeCoNiCuCr. The lowest-energy configuration is identified with a red dashed box.

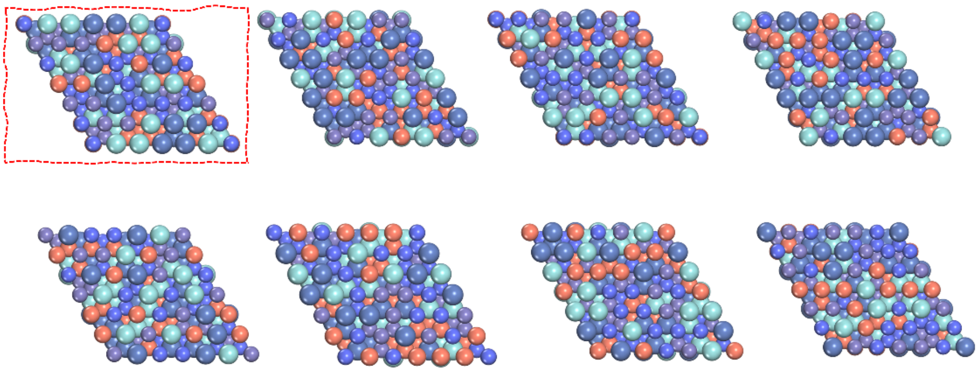

FeCoNiCuZr

Figure S5. Surface configurations of FeCoNiCuZr. The red dashed box indicates the model with the lowest surface energy.

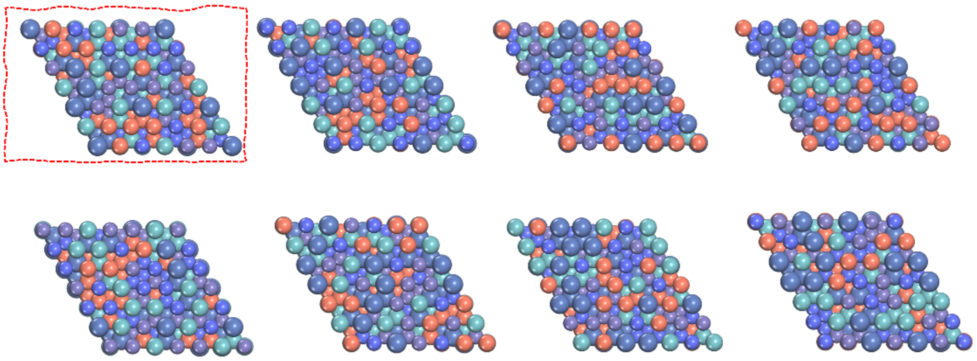

FeCoNiCuNb

Figure S6. Randomly generated FeCoNiCuNb surface structures. The configuration with the lowest surface energy is denoted by a red dashed box.

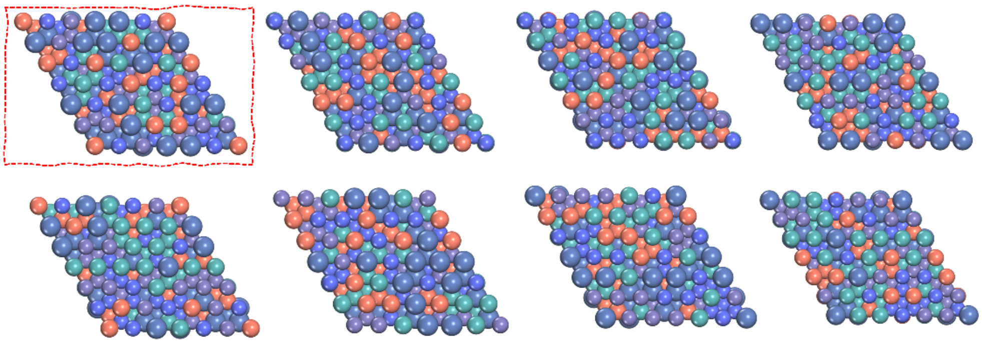

FeCoNiCuMo

Figure S7. Surface models of FeCoNiCuMo. The lowest surface energy configuration is highlighted by the red dashed box.

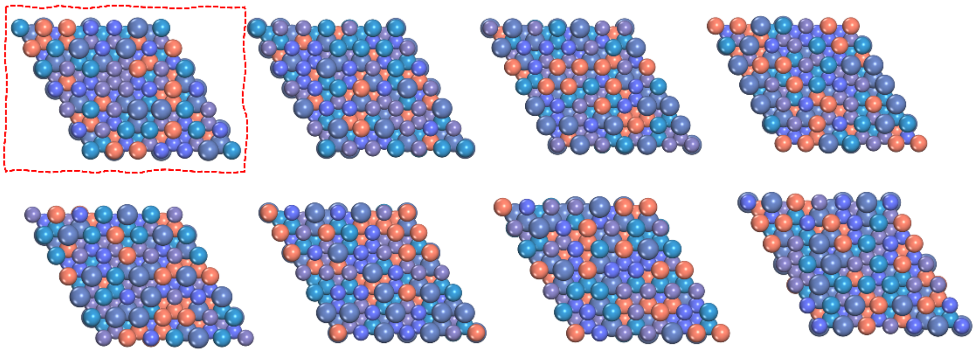

FeCoNiCuW

Figure S8. Random surface configurations of FeCoNiCuW. The red dashed box marks the structure exhibiting the minimum surface energy.

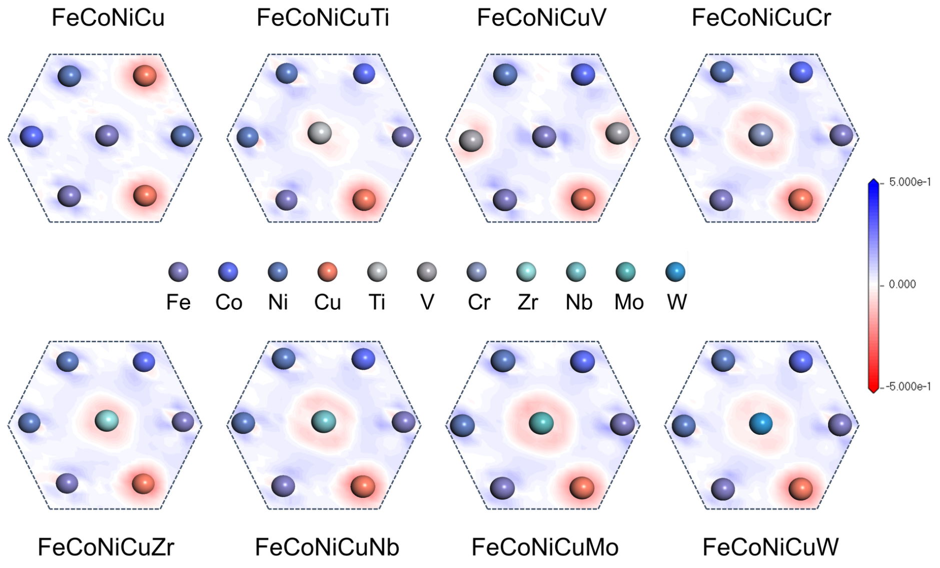

Figure S9. Two-dimensional charge density difference (2D-CDD) maps for various HEA surface atomic configurations, illustrating spatial electron redistribution.

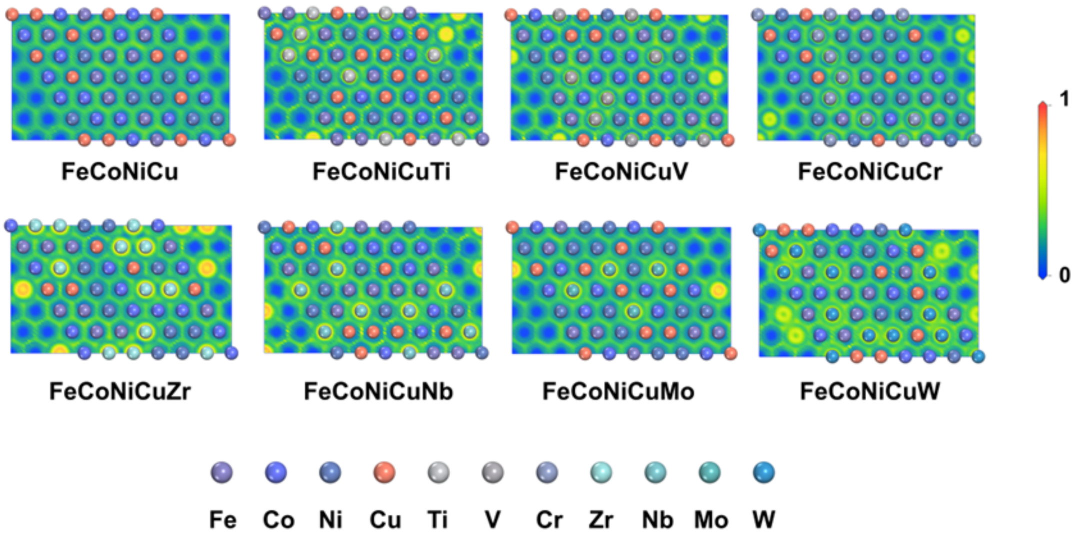

Figure S10. Two-dimensional electron localization function (ELF) maps for FeCoNiCu and FeCoNiCuM models. ELF values range from 0 (delocalized electrons) to 1 (fully localized bonding electrons).

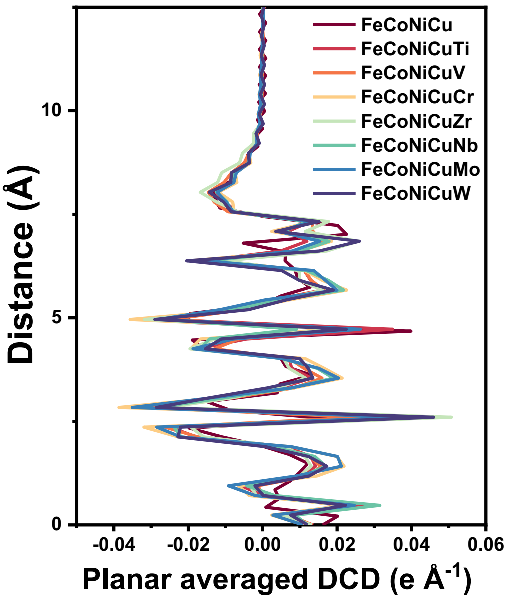

Figure S11. Plane-averaged charge density difference (DCD) profiles along the z-direction of constructed HEA surfaces.

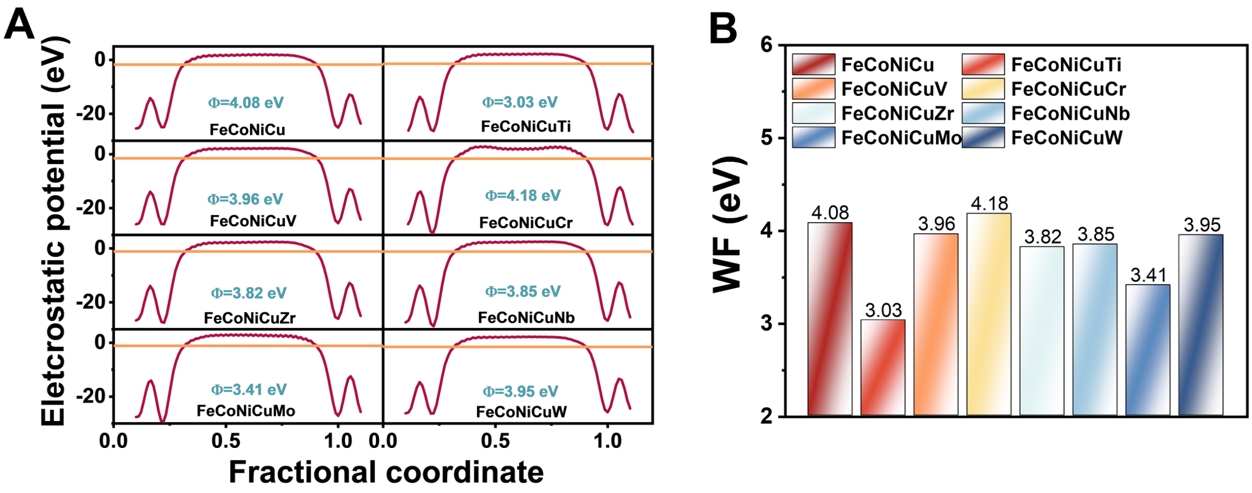

Figure S12. (A) Calculated electrostatic potential maps; (B) Work function (WF) values for different HEA surface models.

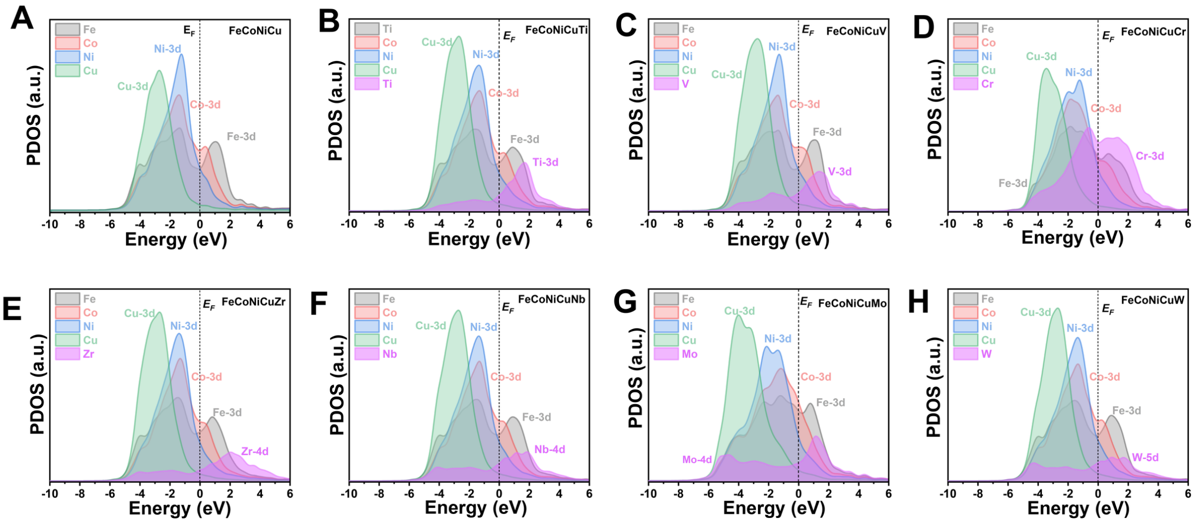

Figure S13. Projected density of states (PDOS) for (A) FeCoNiCu, (B) FeCoNiCuTi, (C) FeCoNiCuV, (D) FeCoNiCuCr, (E) FeCoNiCuZr, (F) FeCoNiCuNb, (G) FeCoNiCuMo, and (H) FeCoNiCuW.

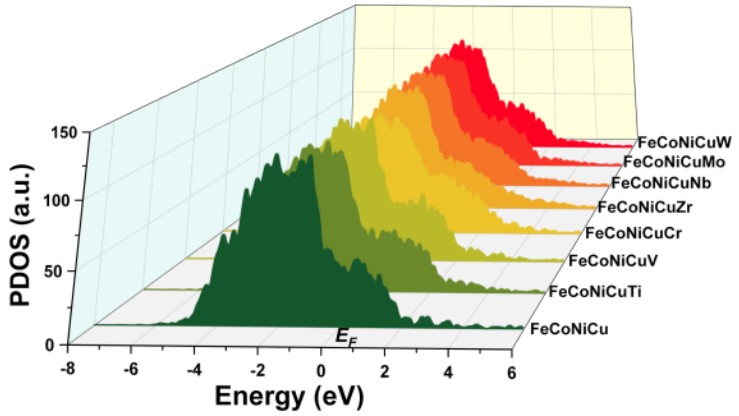

Figure S14. Calculated d-orbital PDOS profiles for various FeCoNiCuM HEA surface models.

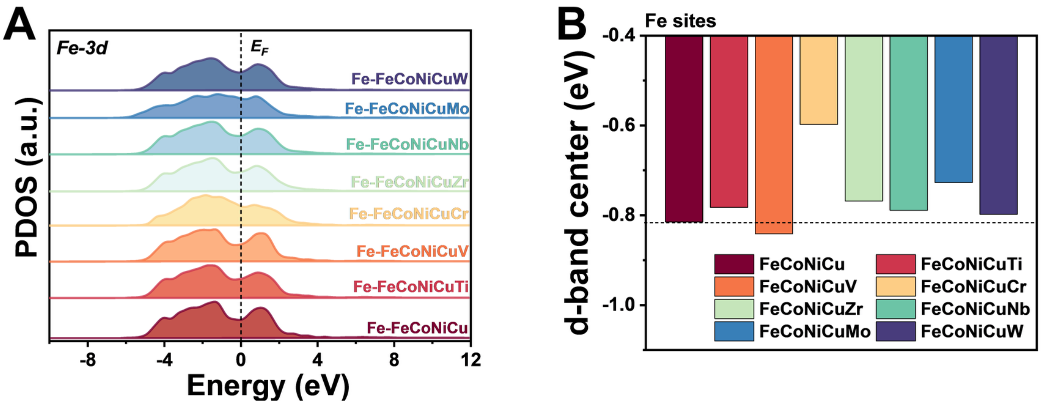

Figure S15. (A) PDOS of Fe atoms in distinct environments. (B) Corresponding d-band center positions of Fe.

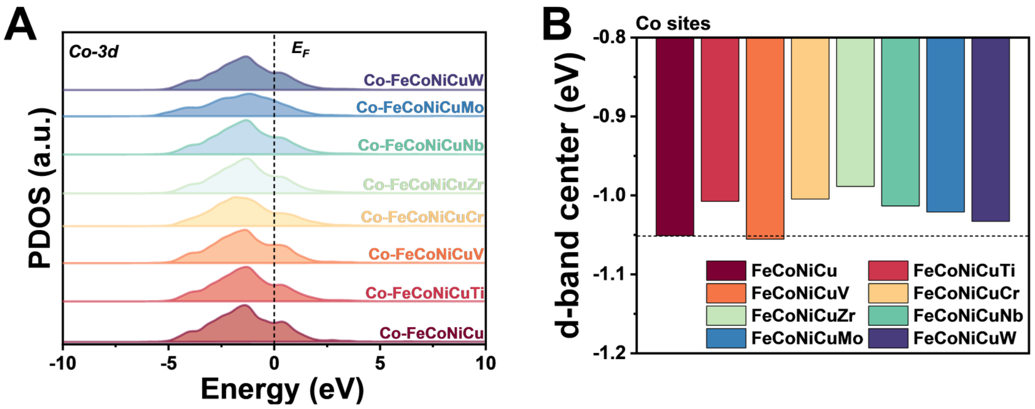

Figure S16. (A) PDOS of Co atoms in different HEA environments. (B) Corresponding d-band center positions of Co.

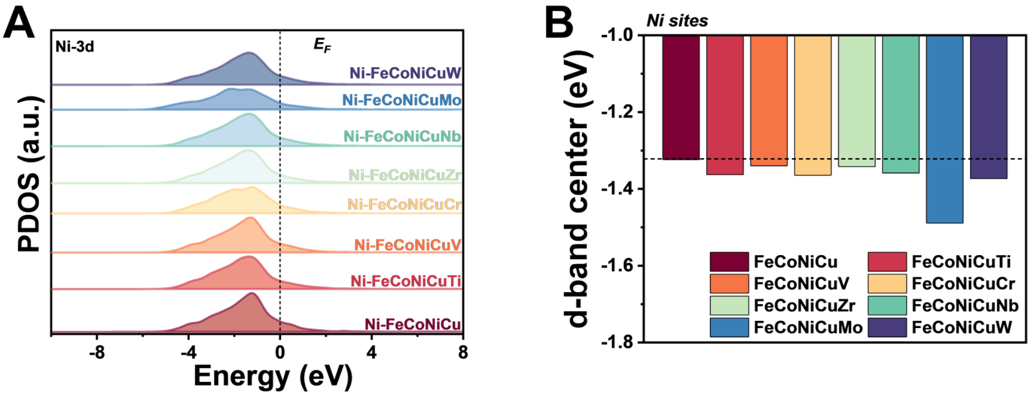

Figure S17. (A) PDOS of Ni atoms in various local environments. (B) Corresponding d-band center positions of Ni

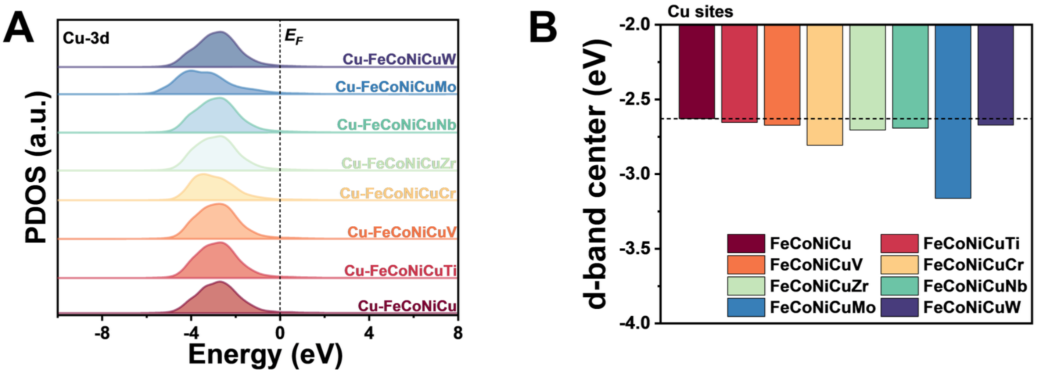

Figure S18. (A) PDOS of Cu atoms in distinct atomic environments. (B) Calculated d-band centers of Cu.

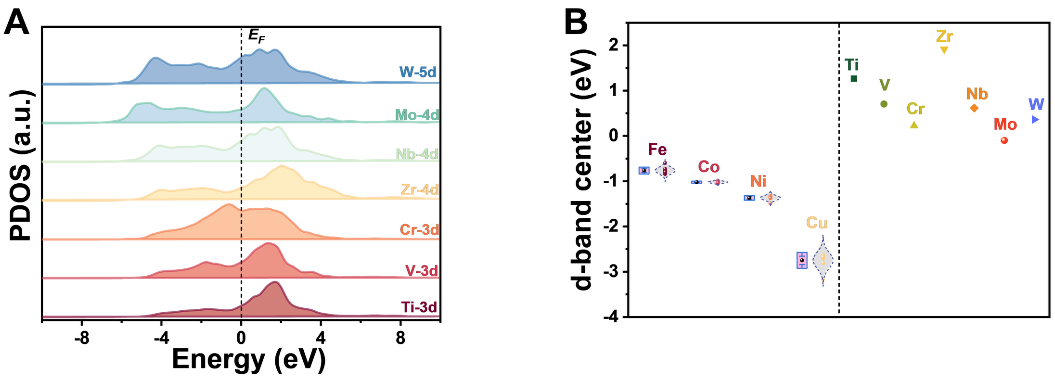

Figure S19. (A) PDOS of Ti, V, Cr, Zr, Nb, Mo, and W atoms in the FeCoNiCuM matrices. (B) Their corresponding d-band centers.

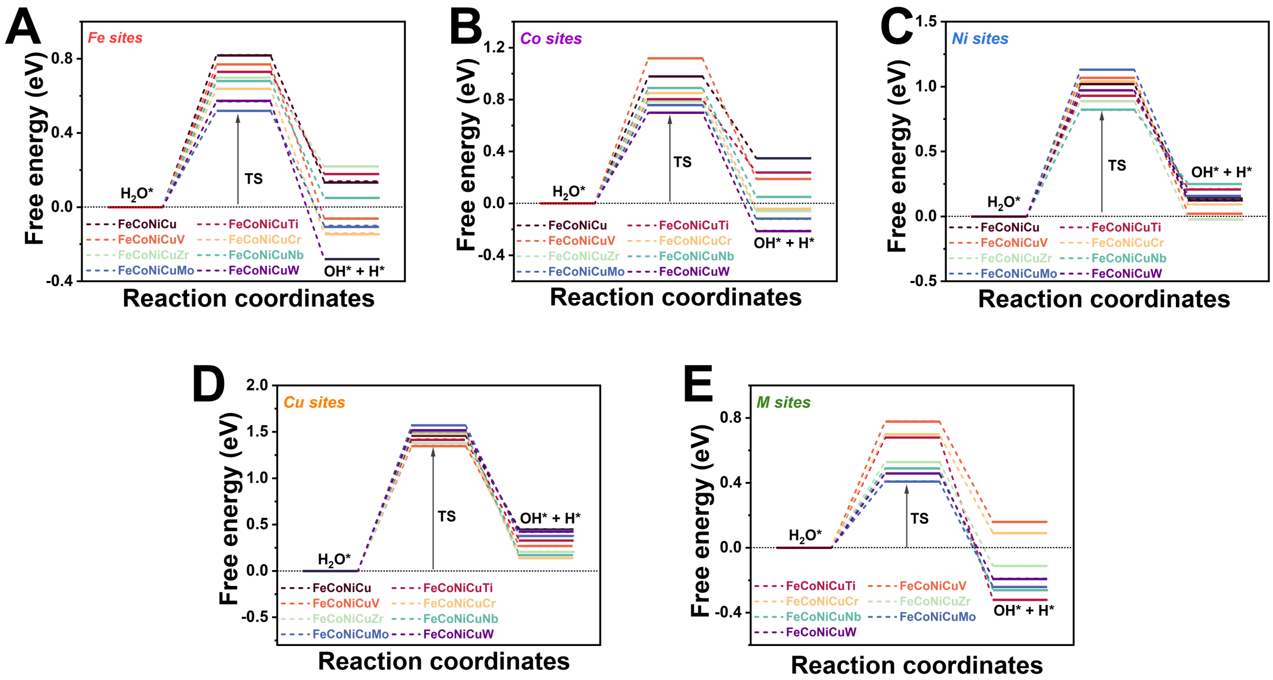

Figure S20. Calculated activation energy barriers for H_2O dissociation at (A) Fe, (B) Co, (C) Ni, (D) Cu, and (E) M sites across the HEA surfaces.

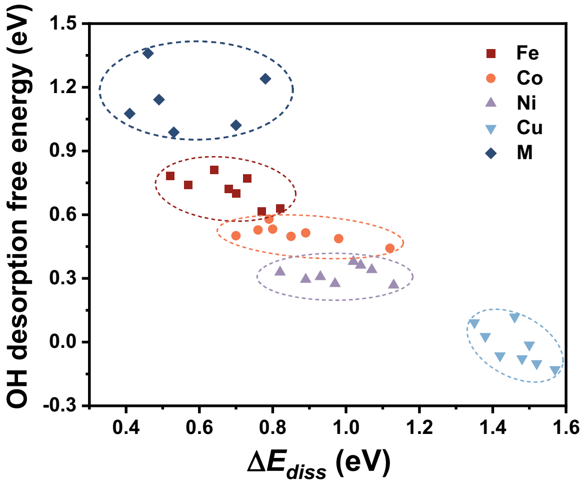

Figure S21. The calculated H_2O dissociation energy (ΔE_{diss}) and OH desorption free energies on various HEA surfaces.

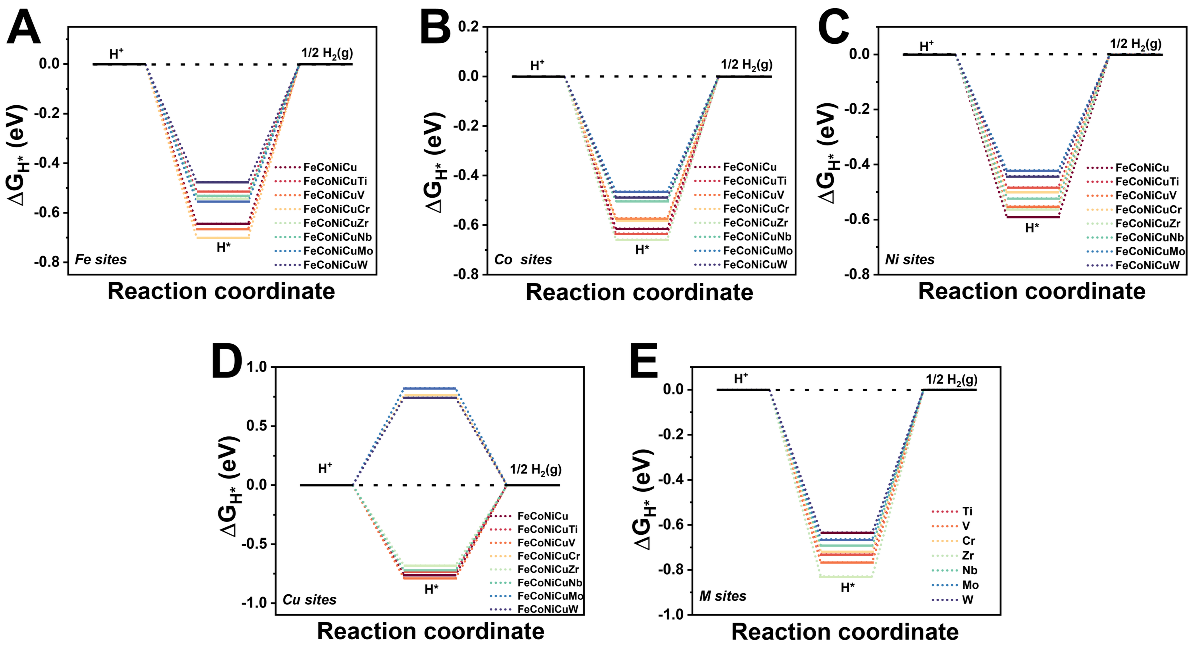

Figure S22. Gibbs free energy of hydrogen adsorption (ΔG_{H^*}) at (A) Fe, (B) Co, (C) Ni, (D) Cu, and (E) M top sites in multiple HEA configurations.

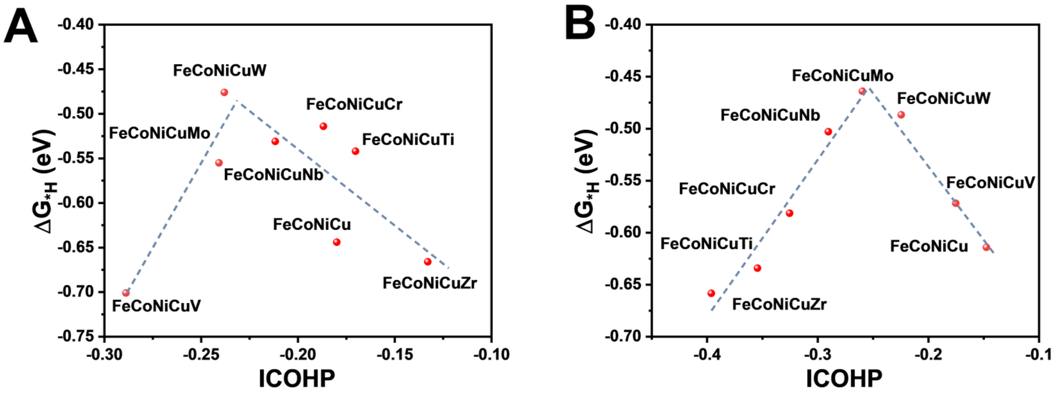

Figure S23. Correlation between ΔG_{H*} and ICOHP values for (A) Fe-H and (B) Co-H bonding configurations.

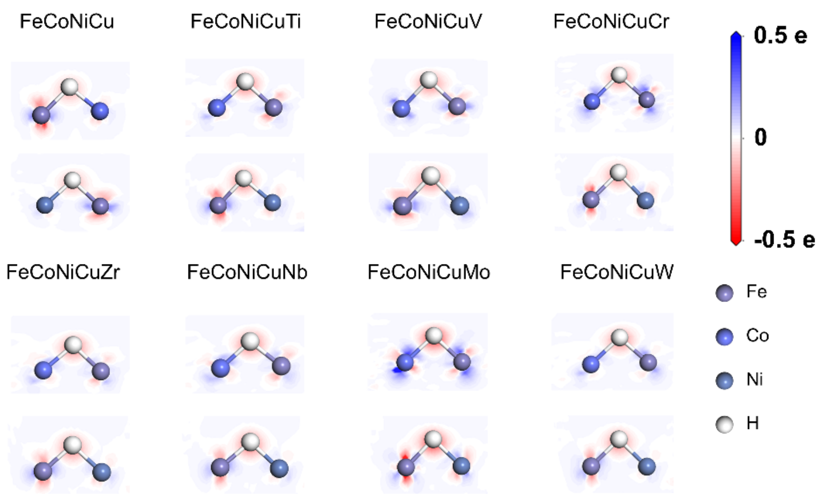

Figure S24. Two-dimensional electron density difference maps for H* adsorption configurations. Blue and red represent electron depletion and accumulation (in units of 0.5 e Å^{-3}), respectively.

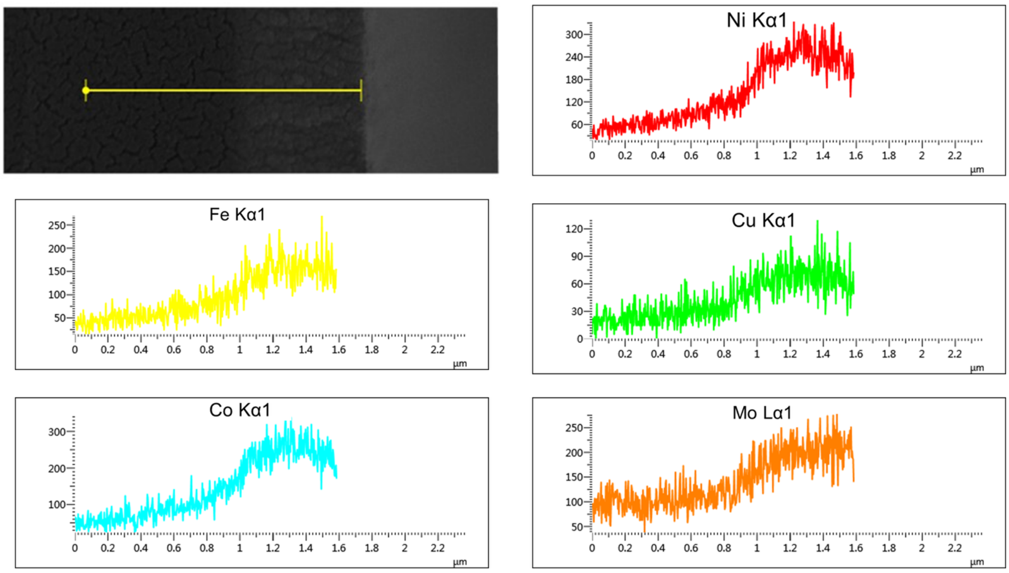

Figure S25. Cross-sectional SEM image and EDS line scans of FeCoNiCuMo films deposited on Si wafer. Film thickness is approximately 500 nm.

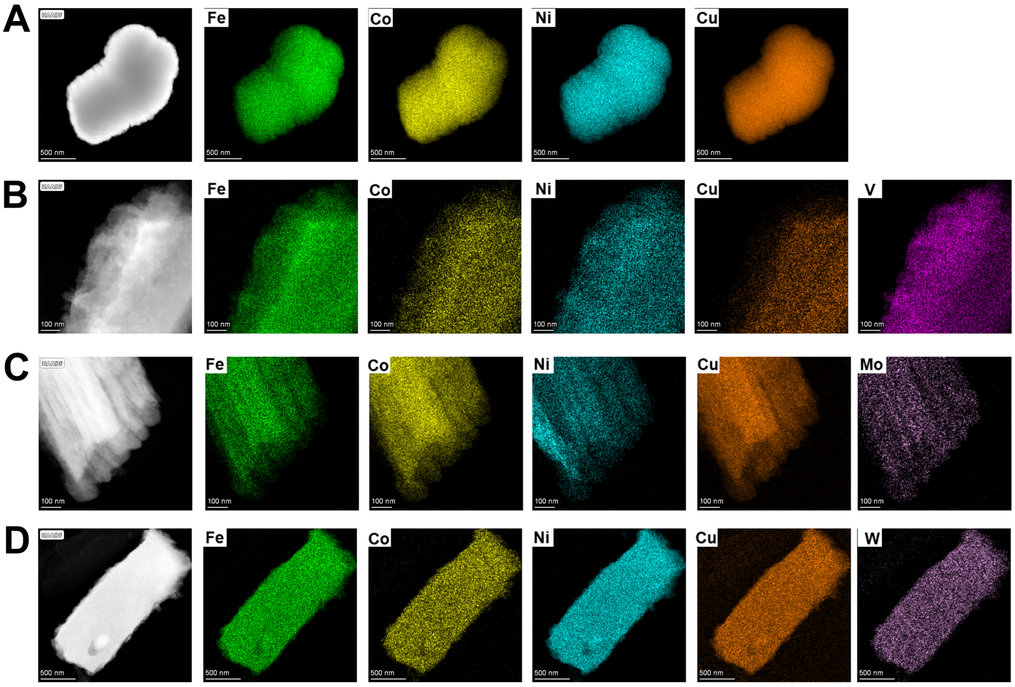

Figure S26. HAADF-STEM images and corresponding elemental EDS maps of (A) FeCoNiCu, (B) FeCoNiCuV, (C) FeCoNiCuMo, and (D) FeCoNiCuW thin films.

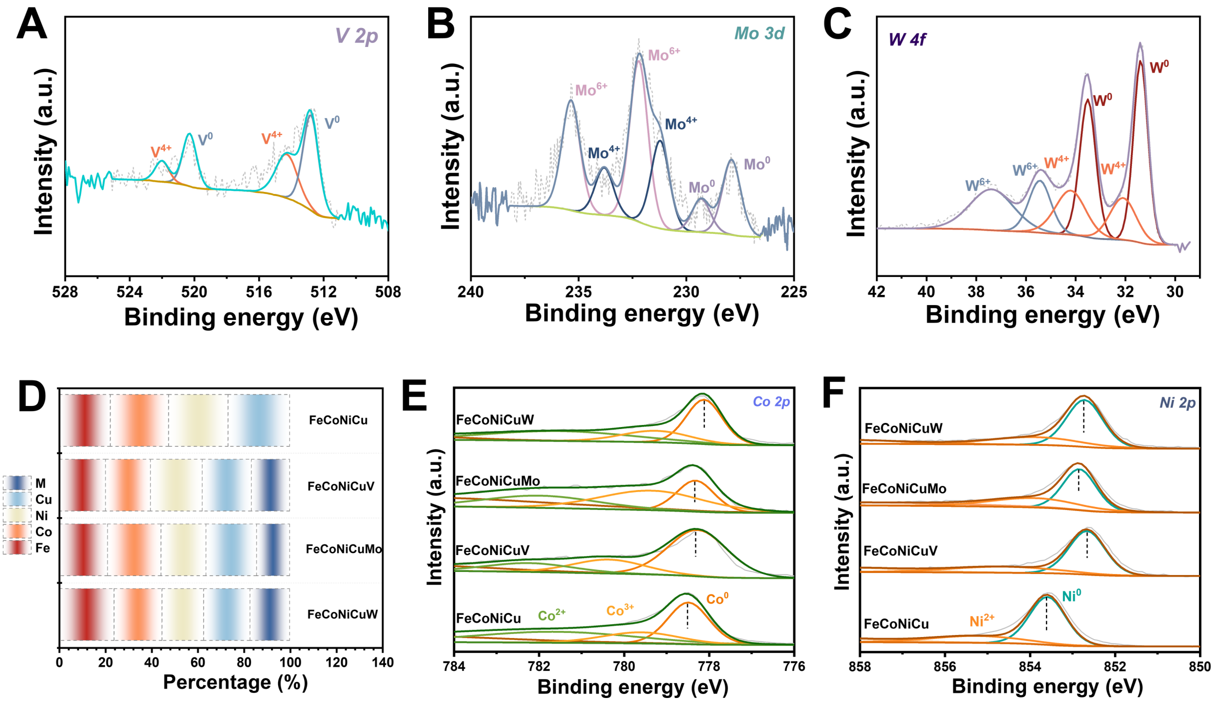

Figure S27. High-resolution XPS spectra of (A) V 2p in FeCoNiCuV, (B) Mo 3d in FeCoNiCuMo, and (C) W 4f in FeCoNiCuW films, (D) Elemental compositions (at.%) determined by XPS spectrum, Enlarged high-resolution XPS spectra of the (E) Co 2p and (F) Ni 2p core levels from **Figure 5** in the manuscript.

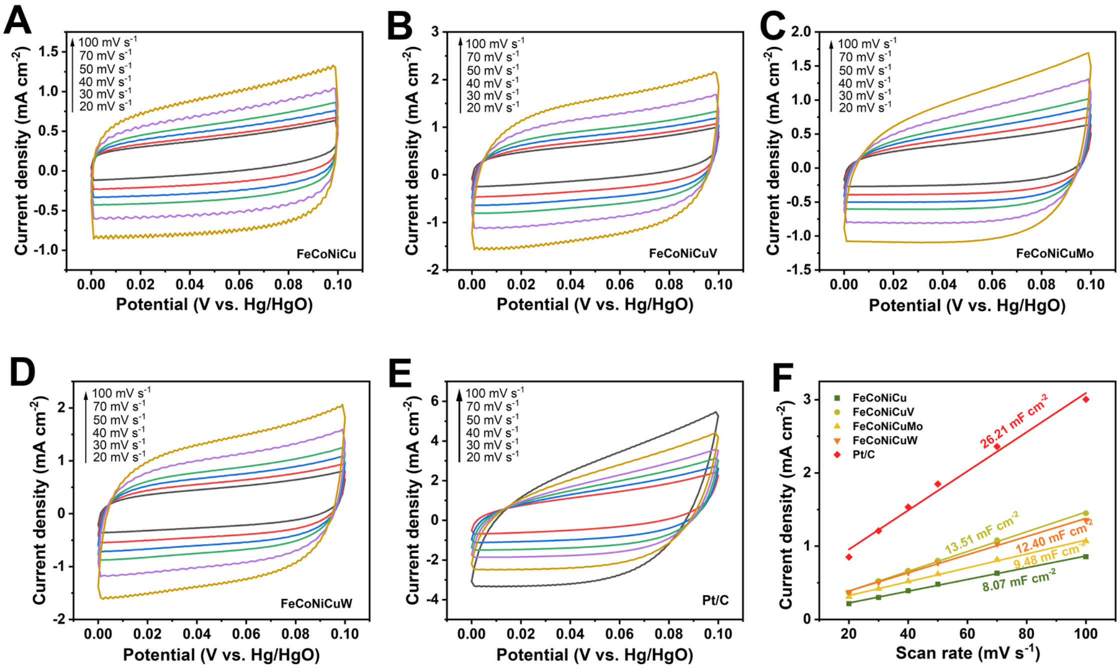

Figure S28. (A–E) Cyclic voltammetry curves of FeCoNiCu-based HEAs and commercial Pt/C at scan rates of 20–100 mV s^{-1}. (F) Calculated double-layer capacitance (C_{dl}) values used to estimate ECSA.

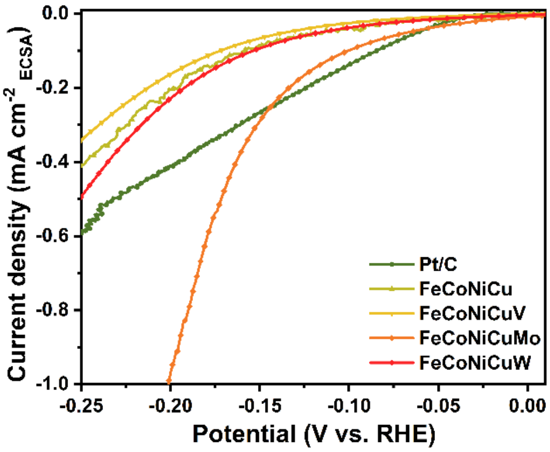

Figure S29. ECSA-normalized LSV polarization curves for various FeCoNiCuM catalysts.

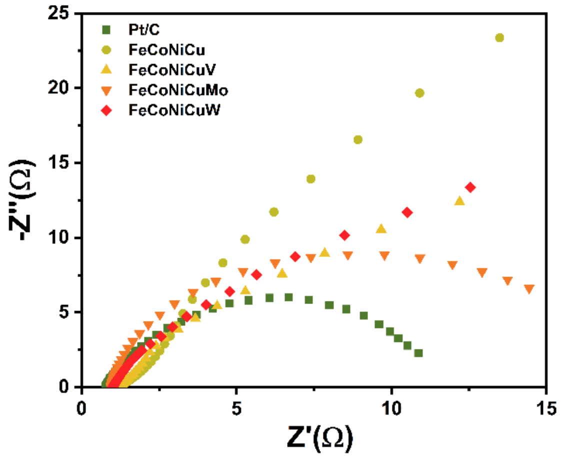

Figure 30. Nyquist plots obtained from electrochemical impedance spectroscopy (EIS) during HER testing of FeCoNiCuM catalysts.

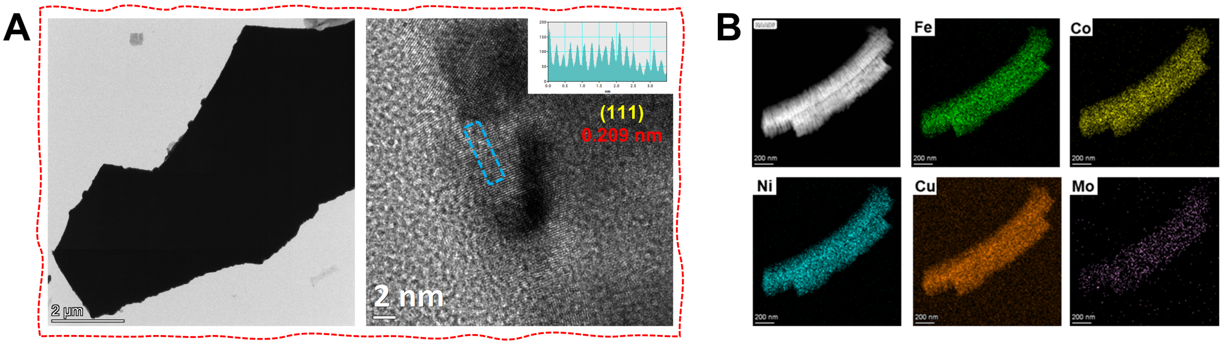

Figure S31. Post-HER structural characterization of FeCoNiCuMo film. (A) TEM and HRTEM images. (B) HAADF-STEM and EDS elemental mapping. Retains *fcc* structure without visible degradation.

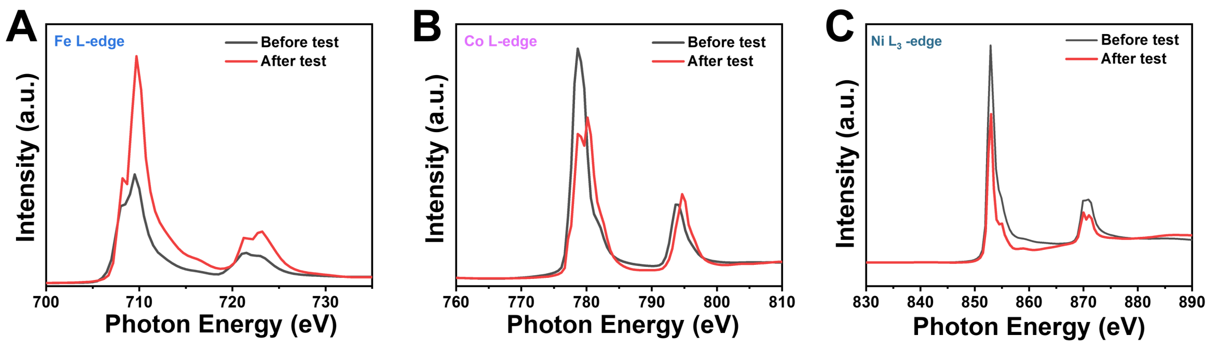

Figure S32. Soft X-ray absorption spectroscopy (sXAS) spectra of FeCoNiCuMo before and after HER: (A) Fe L-edge, (B) Co L-edge, and (C) Ni L-edge.

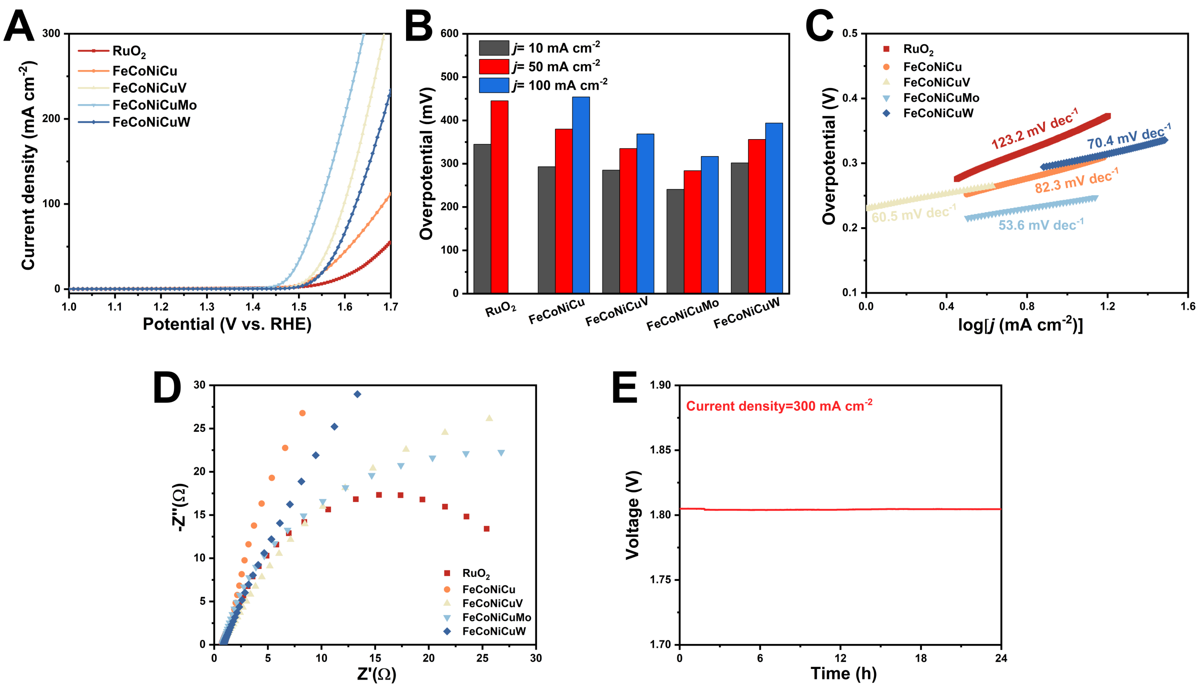

Figure S33. OER performance of FeCoNiCu-based catalysts in 1 M KOH. (A) LSV curves, (B) overpotentials at 10, 50, 100 mA cm^{-2}, (C) Tafel plots, (D) Nyquist plots, and (E) stability via chronopotentiometry.

Table S1. Atomic radii of the constituent elements used in the HEA models.

	**Atomic radius (Å)**
**Fe**	1.26
**Co**	1.25
**Ni**	1.24
**Cu**	1.28
**Ti**	1.47
**V**	1.34
**Cr**	1.28
**Zr**	1.60
**Nb**	1.46
**Mo**	1.39
**W**	1.39

Table S2. Enthalpy of mixing values (kJ mol^{-1}) between HEA elements, indicating mutual miscibility and stability[1].

	**Fe**	**Co**	**Ni**	**Cu**	**Ti**	**V**	**Cr**	**Zr**	**Nb**	**Mo**	**W**
**Fe**		-1	-2	13	-17	-7	-1	-25	-16	-2	0
**Co**	-1		0	6	-28	-14	-4	-41	-25	-5	-1
**Ni**	-2	0		4	-35	-18	-7	-49	-30	-7	-3
**Cu**	13	6	4		-9	5	12	-23	3	19	22
**Ti**	-17	-28	-35	-9		-2	-7	0	2	-4	-6
**V**	-7	-14	-18	5	-2		-2	-4	-1	0	-1
**Cr**	-1	-4	-7	12	-7	-2		-12	-7	0	1
**Zr**	-25	-41	-49	-23	0	-4	-12		4	-6	-9
**Nb**	-16	-25	-30	3	2	-1	-7	4		-6	-8
**Mo**	-2	-5	-7	19	-4	0	0	-6	-6		0
**W**	0	-1	-3	22	-6	-1	1	-9	-8	0	

Table S3. DFT-calculated binding energies (eV) for each metal in FeCoNiCuM HEA models.

	**Fe**	**Co**	**Ni**	**Cu**	**M**
**FeCoNiCu**	-4.52768	-2.23663	-3.90038	-3.30542	
**FeCoNiCuTi**	-5.28234	-2.70359	-3.2106	-3.41988	-6.07519
**FeCoNiCuV**	-2.63945	-1.39234	-1.72709	-1.81022	-2.90952
**FeCoNiCuCr**	-4.60205	-3.05833	-4.10082	-4.32056	-6.4067
**FeCoNiCuZr**	-3.8571	-3.44233	-5.38193	-3.88963	-6.86312
**FeCoNiCuNb**	-4.01412	-2.18312	-3.82623	-2.7553	-6.21762
**FeCoNiCuMo**	-4.7513	-2.14598	-4.19149	-4.39573	-6.58689
**FeCoNiCuW**	-6.14045	-2.59731	-4.21295	-2.89189	-8.54884

Table S4. Calculated dissolution potentials (U_{diss}, in V) of metal atoms in FeCoNiCuM surfaces, indicating electrochemical stability.

	Fe	Co	Ni	Cu	M
**FeCoNiCu**	0.78616	0.73232	0.29889	1.12001	
**FeCoNiCuTi**	0.24444	0.86415	-0.12786	1.11034	-1.731
**FeCoNiCuV**	-0.1639	0.22698	-0.90433	0.31066	-2.31035
**FeCoNiCuCr**	0.83092	1.03338	0.31224	1.54875	-0.96402
**FeCoNiCuZr**	0.42535	1.22517	0.96131	1.36578	-0.97543
**FeCoNiCuNb**	0.71246	0.61377	0.18447	0.72367	-1.09221
**FeCoNiCuMo**	1.26324	0.97129	0.35764	1.57449	-0.10927
**FeCoNiCuW**	1.25809	0.83428	0.38125	0.83092	0.18923

Table S5. Calculated mean square displacemen (S_{MSD}) values (Å^2/ps) for individual atoms in various HEA models.

	**Fe**	**Co**	**Ni**	**Cu**	**M**
**FeCoNiCu**	8.153E-5	-1.528E-6	4.677E-5	5.392E-5	
**FeCoNiCuTi**	0.00141	0.00123	0.00141	0.00133	0.00104
**FeCoNiCuV**	0.00133	0.00121	0.00156	0.00144	0.00196
**FeCoNiCuCr**	-7.122E-6	8.802E-6	-2.728E-5	-2.89E-5	3.639E-5
**FeCoNiCuZr**	0.00216	0.00287	0.0035	0.00367	0.00164
**FeCoNiCuNb**	9.303E-5	-6.168E-5	-3.662E-5	3.969E-8	7.178E-6
**FeCoNiCuMo**	-4.364E-5	-2.143E-5	3.249E-5	-5.307E-5	-4.39E-5
**FeCoNiCuW**	-1.428E-5	-1.125E-5	-2.717E-5	-3.056E-5	-3.774E-5

Table S6. Computed charge transfer (e/atom) for metal elements in different HEA configurations.

	**Fe**	**Co**	**Ni**	**Cu**	**M**
**FeCoNiCu**	0.209	0.039	-0.0075	-0.24	
**FeCoNiCuTi**	0.184	0.0155	-0.0663	-0.287	0.1478
**FeCoNiCuV**	0.188	0.029	-0.053	-0.273	0.29
**FeCoNiCuCr**	0.2118	0.051	-0.02	-0.287	0.12
**FeCoNiCuZr**	0.13	-0.026	-0.109	-0.37	0.375
**FeCoNiCuNb**	0.161	0.0045	-0.07	-0.361	0.2655
**FeCoNiCuMo**	0.1827	0.03	-0.046	-0.334	0.1673
**FeCoNiCuW**	0.216	0.069	-0.0145	-0.256	-0.015

Table S7. Calculated d-band center positions (eV) of metal elements in various HEA environments.

	Fe	Co	Ni	Cu	M
**FeCoNiCu**	-0.81486	-1.05088	-1.32373	-2.6284	
**FeCoNiCuTi**	-0.78252	-1.00752	-1.36288	-2.65361	1.26695
**FeCoNiCuV**	-0.84128	-1.05546	-1.34012	-2.67248	0.70439
**FeCoNiCuCr**	-0.59768	-1.00447	-1.36422	-2.80715	0.22337
**FeCoNiCuZr**	-0.76827	-0.98862	-1.34186	-2.70498	1.91799
**FeCoNiCuNb**	-0.78892	-1.01338	-1.35869	-2.6921	0.61683
**FeCoNiCuMo**	-0.72724	-1.02104	-1.48881	-3.16166	-0.10008
**FeCoNiCuW**	-0.79789	-1.03276	-1.37302	-2.67146	0.36212

Table S8. Water adsorption energies (eV) on Fe, Co, Ni, Cu, and M top sites across FeCoNiCuM HEAs.

	**Fe**	**Co**	**Ni**	**Cu**	**M**
**FeCoNiCu**	-0.421	-0.288	-0.219	0.06	
**FeCoNiCuTi**	-0.456	-0.321	-0.156	0.104	-0.592
**FeCoNiCuV**	-0.363	-0.262	-0.203	0.171	-0.558
**FeCoNiCuCr**	-0.515	-0.318	-0.169	0.212	-0.537
**FeCoNiCuZr**	-0.475	-0.334	-0.191	0.194	-0.629
**FeCoNiCuNb**	-0.442	-0.309	-0.183	0.124	-0.593
**FeCoNiCuMo**	-0.501	-0.313	-0.141	0.265	-0.652
**FeCoNiCuW**	-0.471	-0.302	-0.162	0.178	-0.698

Table S9. OH^- adsorption energies (eV) on various HEA top sites, indicating OH binding strengths.

	**Fe**	**Co**	**Ni**	**Cu**	**M**
**FeCoNiCu**	-0.491	-0.377	-0.315	-0.112	
**FeCoNiCuTi**	-0.523	-0.412	-0.277	-0.094	-0.843
**FeCoNiCuV**	-0.461	-0.345	-0.293	-0.111	-0.724
**FeCoNiCuCr**	-0.551	-0.401	-0.303	0.052	-0.683
**FeCoNiCuZr**	-0.517	-0.425	-0.272	-0.02	-0.826
**FeCoNiCuNb**	-0.502	-0.391	-0.282	0.032	-0.782
**FeCoNiCuMo**	-0.542	-0.408	-0.248	0.112	-0.892
**FeCoNiCuW**	-0.528	-0.389	-0.259	0.091	-0.911

Table S10. Calculated water dissociation energies (eV) at metal top sites in FeCoNiCuM HEAs.

	**Fe**	**Co**	**Ni**	**Cu**	**M**
**FeCoNiCu**	0.82	0.98	1.02	1.46	
**FeCoNiCuTi**	0.73	0.8	0.93	1.42	0.68
**FeCoNiCuV**	0.77	1.12	1.07	1.35	0.78
**FeCoNiCuCr**	0.64	0.85	1.04	1.48	0.7
**FeCoNiCuZr**	0.7	0.79	0.89	1.38	0.53
**FeCoNiCuNb**	0.68	0.89	0.82	1.50	0.49
**FeCoNiCuMo**	0.52	0.76	1.13	1.57	0.41
**FeCoNiCuW**	0.57	0.7	0.97	1.52	0.46

Table S11. Free energies of OH desorption (eV) from HEA surfaces.

	Fe	Co	Ni	Cu	M
**FeCoNiCu**	0.629	0.487	0.379	0.119	
**FeCoNiCuTi**	0.772	0.532	0.308	-0.064	1.24
**FeCoNiCuV**	0.615	0.442	0.341	0.092	1.021
**FeCoNiCuCr**	0.812	0.498	0.362	-0.078	0.988
**FeCoNiCuZr**	0.701	0.579	0.295	0.027	1.142
**FeCoNiCuNb**	0.722	0.514	0.329	-0.013	1.076
**FeCoNiCuMo**	0.782	0.528	0.268	-0.129	1.36
**FeCoNiCuW**	0.741	0.501	0.275	-0.101	1.42

Table S12. Gibbs free energies of hydrogen adsorption (ΔG_{H^*}, eV) at various top sites. Median values are indicated with blue dots.

	**Fe**	**Co**	**Ni**	**Cu**	**M**
**FeCoNiCu**	0.644	0.61407	0.591	0.761	
**FeCoNiCuTi**	0.514	0.63407	0.483	0.733	0.731
**FeCoNiCuV**	0.666	0.57167	0.55167	0.791	0.767
**FeCoNiCuCr**	0.701	0.58125	0.501	0.761	0.719
**FeCoNiCuZr**	0.542	0.6583	0.562	0.681	0.829
**FeCoNiCuNb**	0.531	0.50288	0.52288	0.724	0.692
**FeCoNiCuMo**	0.555	0.46407	0.421	0.822	0.664
**FeCoNiCuW**	0.476	0.48667	0.442	0.742	0.634

Table S13. Standard reduction potentials (E° vs SHE) for the constituent metals used in the HEA design[2].

Reduction in half reaction	E° (V versus SHE)
$Fe^{2+} + 2e^{-} \rightarrow Fe(s)$	-0.44
$Co^{2+} + 2e^{-} \rightarrow Co(s)$	-0.28
$Ni^{2+} + 2e^{-} \rightarrow Ni(s)$	-0.25
$Cu^{2+} + 2e^{-} \rightarrow Cu(s)$	0.337
$Mo(VI) + e^{-} \rightarrow Mo(V)$	0.53
$V^{3+} + e^{-} \rightarrow V^{2+}$	-0.26
$W^{3+} + 3e^{-} \rightarrow W(s)$	-0.10

Table S14. Comparison of overpotentials at 10 mA cm^{-2} and Tafel slopes for HER performance of selected noble metal-based catalysts in 1.0 M KOH, including this work.

Catalyst	**Overpotential, mV** (at 10 mA cm^{-2})	**Tafel slope** (mV dec^{-1})	**Ref.**
**Noble-metal based**			
Ru-$NiPS_3$ NSs	58	64	3
Ru c-SACs	27	32	4
Pt SA-NiSe V	45	52	5
Ni@IrNi	33	32	6
β-$Ni(OH)_2$/Pt	92	51	7
Sr_2RuO_4	61	50	8
$Cu_{2-x}S$@Ru	82	29	9
Mo-$RuCoO_x$	41	42	10
d-$PtSe_2$	59	88	11
Pt−$Co(OH)_2$/CC	32	70	12
FeCoNiCuMo	60.1	72.5	**This work**

Table S15. Comparison of overpotentials at 10 mA cm^{-2} and Tafel slopes for HER performance of selected multiple metal-based catalysts in 1.0 M KOH, including this work.

**Catalyst**	**Overpotential, mV** (at 10 mA cm^{-2})	**Tafel slope** (mV dec^{-1})	**Ref.**
**Multiple metal-based**			
FeCoNi	64	125	13
NiCoWS	70	112	14
$NiFeO_x$@NiCu	70	68	15
H-Fe-CoMoS	137	98	16
$FeNiCoMnVO_x$	89	88	17
$Ni_3ZnC_{0.7}$@CoNiCuFe	97	94	18
(MoWVNbTa)C	156	78	19
AgPdCuAu/NiFeCoAu	85	59	20
La_2(CNMZNL)RuO_6	98	41	21
FeCoNiCrCu	84	62	22
NC-CF-PSFN	186	101.4	23
Ni–Mn_3O_4/NF	91	110	24
NiSe nanowire film	96	120	25
Se-(NiCo)S/OH	103	87.3	26
FeCoNiCuMo	60.1	72.5	**This work**

Reference

[1] A. Takeuchi, A. Inoue, *Mater. Trans.* **2005**, *46*, 2817–2829.

[2] A. J. Bard, R. Parsons, J. Jordan, Standard potentials in aqueous solution. (CRC Press, Boca Raton, 1985).

[3] Q. Fu, L. W. Wong, F. Zheng, X. Zheng, C. S. Tsang, K. H. Lai, W. Shen, T. H. Ly, Q. Deng, J. Zhao, *Nat Commun,* **2023** 14, 6462.

[4] B. Jiang, J. Zhu, Z. Xia, J. Lyu, X. Li, L. Zheng, C. Chen, S. Chaemchuen, T. Bu, F. Verpoort, S. Mu, J. Wu, John Wang, Z. Kou, *Adv. Mater.* **2024**, 36, 2310699.

[5] Z. Chen, X. Li, J. Zhao, S. Zhang, J. Wang, H. Zhang, J. Zhang, Q. Dong, W. Zhang, W. Hu, X.

Han, *Angew. Chem. Int. Ed.* **2023**: e202308686.

[6] J. Xu, X. Wang, X. Mao, K. Feng, J. Xu, J. Zhong, L. Wang, N. Han, Y. Li, *Energy Environ. Sci.*, **2023**, 16, 6120.

[7] X. Yu, J. Zhao, L. Zheng, Y. Tong, M. Zhang, G. Xu, C. Li, J. Ma, G. Shi, *ACS Energy Lett.* **2017**, 3, 237.

[8] Y. Zhu, H. A. Tahini, Z. Hu, J. Dai, Y. Chen, H. Sun, W. Zhou, M. Liu, S. C. Smith, H. Wang, Z. Shao, *Nat. Commun.* **2019,** 10, 149.

[9] D. Yoon, J. Lee, B. Seo, B. Kim, H. Baik, S. H. Joo, K. Lee, *Small* **2017**, 13, 1700052.

[10] Y. Zhang, R. Lu, C. Wang, Y. Zhao, L. Qi, *Adv. Funct. Mater.* **2023**, 33, 2303073.

[11] Y. Chang, P. Zhai, J. Hou, J. Zhao, J. Gao, *Adv. Energy Mater* **2022**, 12, 2102359.

[12] Z. Xing, C. Han, D. Wang, Q. Li, X. Yang, *ACS Catal.* **2017**, 7, 7131.

[13] Q. Zhang, N. M. Bedford, J. Pan, X. Lu, R. Amal, *Adv. Energy Mater.* **2019**, 9, 1901312.

[14] M. Ma, J. Xu, H. Wang, X. Zhang, S. Hu, W. Zhou, H. Liu, *Appl. Catal. B-Environ.* **2021**, 297, 120455.

[15] Y. Zhou, Z. Wang, Z. Pan, L. Liu, J. Xi, X. Luo, Y. Shen, *Adv. Mater.* **2019**, 31, 1806769.

[16] Y. Guo, X. Zhou , J. Tang, S. Tanaka, Y. V. Kaneti, J. Na, B. Jiang, Y. Yamauchi, Y.o Bando, Y. Sugahara, *Nano Energy* **2020**, 75, 104913.

[17] S. Ding, Y. Sun, F. Lou, L. Yu, B. Xia, J. Duan, Y. Zhang, S. Chen, *J. Power Sources* **2022**, 520, 230873.

[18] Y. Wang, H. Yang, Z. Zhang, X. Meng, T. Cheng, G. Qin, S. Li, *J. Mater. Sci. Technol.* **2023**, 135, 26-33.

[19] S. Niu , Z. Yang, F. Qi, Y. Han, Z. Shi, Q. Qiu, X. Han, Y. Wang, X. Du, *Adv. Funct. Mater.* **2022**, 32, 2203787.

[20] H. Yang, X. Guo, R. Chen, Q. Liu, J. Liu, J. Yu, C. Lin, J. Wang, M. Zhang, *Appl. Surf. Sci.*

2022, 36, 154808.

[21] N. Sun, Z. Lai, W. Ding, W. Li, T. Wang, Z. Zheng, B. Zhang, X. Dong, P. Wei, P. Du, Z. Hu, C. Pao, W. Huang, H. Wang, M. Lei, K. Huang, R. Yu, *Adv. Sci*. **2024**, 11, 2406453.

[22] Y. Wang. H. Yang, Z. Zhang, X. Meng, T. Cheng, G. Qin, S. Li, *J. Mater. Sci. Technol.* **2023**, 166, 234.

[23] B. Wang , F. Yang , Y. Dong , Y. Cao , J. Wang , B. Yang , Y. Wei , W. Wan , J. Chen , H. Jing, *Chem. Eng. J.* **2020**, 399, 125779.

[24] X. Li, P. Liu, L.Zhang, M. Zu, Y. Yang, H. Yang, *Chem. Commun.* **2016**, 52, 10566.

[25] C. Tang, N. Cheng, Z. Pu, W. Xing, X. Sun, *Angew. Chem. Int. Ed.* **2015**, 54, 9351.

[26] C. Hu, L. Zhang, Z. Zhao, A. Li, X. Chang, J. Gong, *Adv. Mater.* **2018**, 30, 1705538.
